# Appealing to superlative clauses: Or how to split the scope of superlative adjectives across intensional verbs

**DOI:** 10.1007/s11049-025-09686-0

**Published:** 2025-11-11

**Authors:** Isabelle Charnavel

**Affiliations:** Département de Linguistique, Faculté des Lettres, Université de Genève, 5 rue De-Candolle, CH-1211 Genève 4, Switzerland

**Keywords:** Superlative, Degree clause, Comparative, Intensionality, Ellipsis, NPI

## Introduction

The general goal of this article is to defend the hypothesis that comparative clauses have superlative counterparts, which I will call the superlative clause hypothesis. While comparative clauses have been an extensive object of study, superlative clauses are only envisioned in highly restricted cases by a handful of studies. The present paper further argues for the existence of superlative clauses on the basis of cases involving an interaction between intensionality and superlatives. More generally, the argumentation (in the spirit of Gawron 1995) is guided by the hypothesis that due to their morphosyntactic and semantic similarities, comparative and superlative constructions should be given more parallel treatments than they have been so far.

In languages like English, comparison constructions involve a standard of comparison that can be expressed as a comparative clause (clausal comparative as in (1)a) or as a phrase (phrasal comparative as in (1)b) as reviewed in Lechner (2020), i.a.

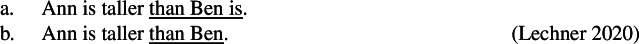

Comparative clauses are standardly treated as degree clauses complementing a two-place degree quantifier -*er*. For example, the *than*-clause in (1)a is analyzed as denoting the degree of Ben’s tallness, which is compared to the degree of Ann’s tallness expressed by the matrix clause. Phrasal comparatives are either treated as elided versions of clausal comparatives as in (2)a (reduced clause analysis as in Heim 1985; Lechner 2001, i.a.) or as simple DPs as in (2)b (direct analysis as in Hankamer 1973; Kennedy 2009, i.a.; cf. Bhatt and Takahashi 2011).^1^

(2)
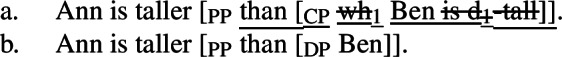

Under the approach in (2)a, all *than*-complements uniformly denote degrees and complement the same two-place degree quantifier -*er* taking two degree arguments to be compared. Under the approach in (2)b, only *than*-clauses denote degrees; *than*-phrases denote individuals, which requires postulating a different, three-place -*er* taking two individual arguments to be compared with respect to a property.

Superlative constructions express comparison in a similar way to comparative constructions, to which they are clearly related morphosyntactically. As emphasized by Gawron (1995) or Loccioni (2019), the only specificity of superlatives is to establish a comparison within a *set* (the domain of comparison) while comparatives relate only two elements. For instance, the degree of Ann’s tallness is evaluated with respect to the tallness of all relevant individuals in (3), while it is compared with only Ben’s in (1).

(3) Ann is (the) tallest (of all).^2^

Despite their similarity, there is no consensus on a parallel analysis of superlatives and comparatives: notwithstanding their divergences, almost all analyses of superlatives treat the domain of comparison as a set of individuals (implicit or expressed by a partitive phrase or a NP) complementing a three-place -*est* (see Heim 1985, 1995/1999, von Fintel 1999; Farkas and Kiss 2000; Matushansky 2008; Krasikova 2012; Loccioni 2018, i.a.); there is virtually no discussion on a superlative counterpart of two-place -*er* and (elided) comparative clauses as in (1)a or (2)a. For example, (4) is standardly assumed to convey that the mountain climbed by John is compared with other mountains (with respect to their height).

(4) John climbed the highest mountain. (Heim 1995/1999)

The superlative morpheme -*est* is thus claimed to take three arguments – an individual (John’s mountain), an (implicit) domain of comparison (all contextually relevant mountains), and a gradable predicate (*high* (*mountain*)) in parallel to three-place -*er*.

There are nevertheless a few exceptions. First, one of Heim’s (1995/1999) analyses of the so-called relative reading of superlatives uses a superlative counterpart of two-place -*er* requiring a degree-based domain of comparison (see also Gawron 1992, 1995 in a different framework^3^). As will be discussed, superlatives like (4) give rise not only to absolute readings (John climbed the highest of all mountains, i.e., Mount Everest), but also to relative readings (John climbed a higher mountain than anyone else did), which gave rise to various analyses diverging with respect to the scope of -*est*, definiteness, and the nature of the comparison class (see Heim 1985, Szabolcsi 1986, Gawron 1995, Heim 1995/1999, Sharvit and Stateva 2002; Romero 2011, i.a.). One of Heim’s (1995/1999) analyses of the relative reading treats the domain of comparison as a set of degree properties determined by focus. Under this view, (4) basically expresses a comparison between the maximal degree of height of the mountain climbed by John and that of mountains climbed by other relevant climbers. The superlative morpheme -*est*, which takes two degree arguments, thus parallels two-place -*er* (see Romero 2011, Tomaszewicz-Özakin 2020).

Second, two independent studies (Romero 2013; Howard 2014) propose that this degree-based domain of comparison can be syntactically expressed as a clause in some restricted configurations such as (5)a and (5)b.

(5)
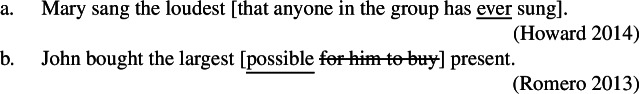

Howard (2014) defends this hypothesis for cases in which a superlative seems to be modified by a relative clause matching the matrix clause and including a negative polarity item (NPI) as in (5)a (see discussion in Sect. 2.2.2). Romero (2013) independently supports a similar hypothesis to account for modal superlative readings such as (5)b (i.e., John bought as large as possible a present for him to buy).

In order to test the default hypothesis that the comparandum should be of the same nature in comparative and superlative constructions, the goal of the present article is to motivate the existence of superlative clauses in further constructions than (5)a and

b. Specifically, we will focus on two empirical cases involving superlatives and intensional predicates, namely so-called ‘intensional superlatives’ (Bhatt and Sharvit 2005) as in (6)a and ‘upstairs *de dicto* readings’ (Sharvit and Stateva 2002) as in (6)b, which remain poorly understood.

(6)
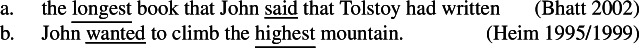

As we will see, both cases present a reading that cannot be straightforwardly derived by standard theories of superlatives. These two puzzles are usually examined separately, and the various solutions that have been proposed for each do not usually extend to the other (see Bumford and Sharvit 2022 for a recent case). Instead, I will hypothesize that the two problems can be given the same solution because they are of the same nature: both cases require the intensional predicate (e.g., *said*, *wanted*) to scope between the superlative morpheme (-*est*) and the gradable predicate (e.g., *long*, *high*) as suggested by Sharvit (2003) and Hulsey and Sauerland (2006) for intensional superlatives and proposed by Heim (1995/1999) for upstairs *de dicto* readings. Such split scope can be uniformly achieved in both cases under the hypothesis that they involve (elided) superlative clauses as roughly represented in (7).

(7)
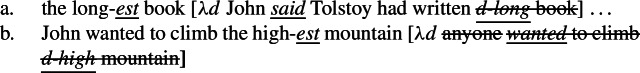

The outline of the rest of the article is as follows. Section 2 concentrates on intensional superlatives like (7)a and shows how the superlative clause hypothesis provides a novel solution deriving all the properties of their so-called low reading. Section 3 focuses on upstairs *de dicto* readings like (7)b and similarly argues that assuming the existence of (elided) superlative clauses allows us to improve on previous analyses. Section 4 concludes.

## Intensional superlatives

Intensional superlatives (as dubbed by Bhatt and Sharvit 2005) are superlative adjectives such as *longest* in (8) (repeating (6)a) that seem to modify the head of a relative clause containing an intensional predicate such as *said*.

(8)
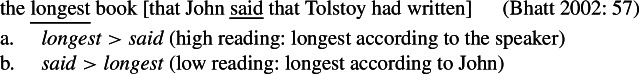

As first observed by Bhatt (2002), (8) seems to exhibit two readings depending on who is understood as evaluating the length of the book – John or the speaker. In the latter case (i.e., (8)a), John only expresses an opinion about the authorship of the book. Under this interpretation, (8) may for instance denote “War and Peace” if the speaker and John are not mistaken about book length and book authorship, respectively. This reading is called the high reading because the superlative adjective *longest* seems to be interpreted above the intensional predicate *said*. In the former case (i.e., (8)b), John further expresses an opinion about the length of the book, which is reported by the (possibly disagreeing) speaker. Under this construal, (8) may for example refer to “Anna Karenina” if John is mistaken about book length, but not about book authorship. This reading is called the low reading because *longest* seems to be interpreted below *said*.

According to Bhatt (2002), these two readings reveal two scopal possibilities, which provide a new argument for the raising analysis of relative clauses. Specifically, he argues that the low reading derives from reconstruction of the superlative adjective within the relative clause. Under the low reading, *longest book* in (8) must be interpreted within the relative clause, just like the part of the idiom *headway* in (9). This is only possible, so the argument goes, if the head of the relative clause originates internal to the relative clause.

(9)
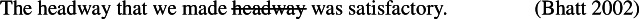

As we will briefly review in Sect. 2.1, Bhatt’s reconstruction hypothesis remains debated because it faces several challenges; but alternative existing hypotheses are not without their problems either. In Sect. 2.2, we will see that the superlative clause hypothesis provides a novel solution that reconciles both sides of the debate. The core idea consists in treating the bracketed clause in (8) under the low reading as a superlative clause, which parallels the comparative clause in (10).

(10)
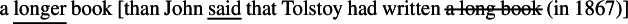

This hypothesis entails split scope of the superlative morpheme -*est*, which, like -*er*, is interpreted outside the clause, and the gradable predicate *long*, which is interpreted within the clause (see (7)a). Such split scope, I will argue, is the key to reconciling the arguments for and against the reconstruction hypothesis.

As mentioned above, superlatives nevertheless differ from comparatives in partitivity: the comparison must be made within a set, which entails that the domain of comparison must contain what I will call the correlate (the clause in which -*est* appears^4^). This difference will motivate ellipsis of a clause identical to the superlative clause as roughly represented in (11).

(11)

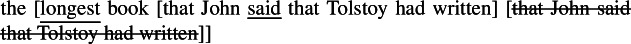

We will further see that some aspects of the problem are complicated by some confusion about the description of the readings in the literature. I will argue that some instances of the so-called high reading (e.g., with high *ever* as in *the longest book that John* *ever* *said that Tolstoy had written*) should in fact be analyzed as a variant of the low reading, in the sense that the measuring relation (e.g., length) is still evaluated by the attitude holder (e.g., John) in this case, although the speaker is responsible for making the comparison between those lengths.

### The debate on superlative reconstruction

There are two main types of approaches to derive the low reading of intensional superlatives in the literature: those − like Bhatt’s − that rely on reconstruction, and those that do not. Besides interpretation, the main arguments fueling the debate involve NPI licensing, intervention effects and the specificity of superlatives as compared to other modifiers. But as briefly reviewed in Sects. 2.1.1 and 2.1.2 (see further details in Charnavel 2022), both types of account face outstanding problems, which I will argue in Sect. 2.2 that the superlative clause hypothesis can solve, as previewed in Table 1.

**Table 1:** The main points of the debate on intensional superlative [*sign indicates problems]

	Reconstruction hypothesis *(Bhatt* 2002*; Bhatt and Sharvit* 2005*, Hulsey and Sauerland* 2006*, i.a.)*	Neg-raising hypothesis *(Heycock* 2005*,* 2019*, cf. Sharvit* 2003*)*	Superlative clause hypothesis
Interpretation: derivation of low reading	Reconstruction of *longest* *overgeneration of some low readings	*Neg*-lowering of superlative entailment *overgeneration of some low readings	Split scope of -*est* and measuring relation; superlative clause
Low NPI licensing	NPI licensing by reconstructed superlative *undergeneration of intervention effects	NPI licensing by nonreconstructed superlative	NPI licensing in superlative clauses
Intervention effects for low reading	A-bar movement of superlative *undergeneration of all intervention effects	Low reading only with neg-raising predicates *overgeneration of intervention effects with some nonneg-raising predicates like *say* *undergeneration of intervention effects with some neg-raising predicates like *should*	Intervention effects due to degree quantification
Other modifiers	Low reading with modifiers that can reconstruct *overgeneration of low readings for other modifiers than superlatives	Low reading with modifiers inducing negative entailment	Low reading with superlative modifiers, i.e., taking a degree clause as domain of comparison

#### Reconstruction account

Bhatt’s reconstruction account is mainly motivated by interpretative aspects of intensional superlatives. First, as mentioned, they induce a so-called low reading under which, Bhatt claims, they scope under the intensional verb within the relative clause. Second, the two readings of intensional superlatives, Bhatt argues, can be disambiguated by inserting NPIs in high or low positions, as in (12).

(12)
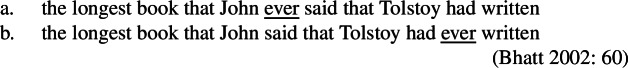

According to Bhatt (2002) (cf. Bhatt and Sharvit 2005), (12)a involving *ever* in the higher clause only exhibits the high reading, and (12)b involving *ever* in the lower clause only exhibits the low reading. This correlation, he claims, derives from locality constraints on NPI licensing: the superlative *longest* can only license the NPI *ever* if it is interpreted within the same clause as *ever*; in (12)b, this can only obtain under reconstruction.

Hulsey and Sauerland (2006) add a third argument to Bhatt’s type of analysis. They argue that as predicted by a reconstruction account, not just superlatives, but any adjective, such as *wonderful* in (13), can trigger a low reading: (13) can refer to books judged to be wonderful by Siouxsie, but not by the speaker.

(13) The wonderful books that Siouxsie said that Lydia had written (Hulsey and Sauerland 2006: 125)

But all three arguments are challenged by Heycock (2005, 2019) and Sharvit (2003). First, Heycock (2005) points out that the assumption on which Bhatt’s argument relies is incorrect: weak NPIs like *ever* do not require a clausemate licensor: both high and low NPIs are licensed in noun complement clauses of superlatives as in (14) (where reconstruction is impossible, unlike in relative clauses).

(14)
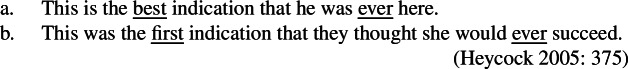

Therefore, sentences like (12)b do not constitute evidence for reconstruction, after all. This conclusion is further supported by the absence of correlation between the low reading and binding conditions as shown in Heycock (2019). Moreover, Heycock (2005) shows that intervention effects for NPI licensing provide direct evidence against reconstruction.

(15)
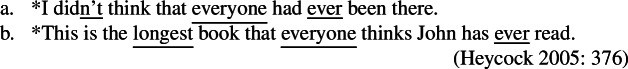

Universal quantifiers like *everyone*, which are independently known to intervene for NPI licensing as illustrated in (15)a, induce intervention effects with intensional superlatives as in (15)b. But *everyone* could not intervene if *longest* had reconstructed below *thinks* as hypothesized by Bhatt.

Second, Sharvit (2003) shows that assuming reconstruction of the superlative overgenerates readings under any semantics of the superlative morpheme -*est*. Bhatt (2002) uses Heim’s (1995/1999) two-place -*est* in (16), which involves a degree-based comparison set, to derive the low reading.

(16)
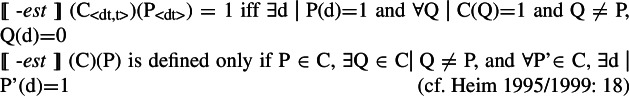

This lexical entry, which requires -*est* movement, takes two arguments: an implicit, focus-based domain argument C consisting of a set of degree properties determined via association with focus (Rooth 1992), and a property of degrees P. As mentioned in the introduction, Heim (1995/1999) specifically designs it to capture relative readings of superlatives, as illustrated in (17).

(17)
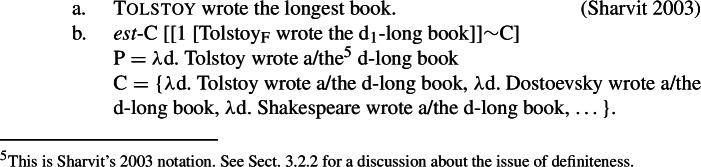

The relative reading of (17)a, which is favored by focusing *Tolstoy* (as indicated by small capital letters) implies a comparison between book authors. This is derived under (16) by making -*est* focus sensitive: -*est* moves to a propositional level and takes as first (implicit) argument C the focus value of its complement P. (17)b thus predicts (17)a to be true if and only if there is a degree such that Tolstoy wrote a book long to that degree, and no other relevant author wrote a book to that degree.

To derive the low reading as shown in (18), *longest* must reconstruct in the relative clause, move to the edge of the embedded clause and focus-associate with the trace of the relative head (or more precisely, with the embedded variable in the lowest copy of the head after trace conversion à la Fox 2002).

(18)
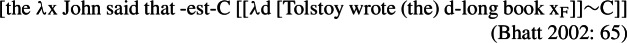

This proposal correctly derives the low reading. But as demonstrated by Sharvit (2003), it overgenerates unless it is stipulated that only the variable inside the trace in the scope of -*est* can be focused. In particular, the interpretation under which John said (17)a is unavailable even with focus on *Tolstoy* as in (19) (in this case, Tolstoy is contrasted with other individuals as authors mentioned by John, not as authors of the longest book). But Bhatt’s derivation predicts it to be available as long as -*est* focus-associates with *Tolstoy* as is the case in (19)b.

(19)
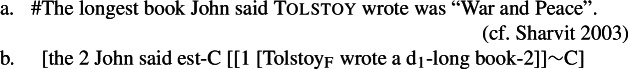

Sharvit (2003) further shows that the problem is even worse with 3-place -*est*, which involves an individual-based domain: no value of the comparison set can give rise to the low reading (unless some stipulations are made, as in Hulsey and Sauerland 2006). Under Bhatt’s account, the two main lexical entries for -*est* thus incorrectly predict the existence of a reading implying a comparison between books by Tolstoy and books by other contextually relevant authors.

Third, Heycock (2005) shows that the seemingly low reading of nonsuperlative adjectives like *wonderful* exhibits specific properties suggesting that this reading is not derived in the same way: to give rise to this reading, nonsuperlative modifiers are associated with a scare quote intonation; they do not require intensional operators (see (20)); and they are not subject to intervention effects (see (21)).

(20) Siouxie was always going on about the books that Lydia had written. But I’ve read those wonderful books and they are complete rubbish. (Heycock 2005: 362)

(21) The expensive car that his wife didn’t think he should buy was actually a Ford Mondeo. (Heycock 2005: 363)

By contrast, Heycock (2005) shows that the low reading of intensional superlatives is blocked by intervening elements such as the negation, as illustrated in (22).

(22) # the longest book that John didn’t say that Tolstoy had written

Given all this evidence against reconstruction, Heycock (2005, 2019) proposes an alternative account motivated by such intervention effects as reviewed in the next section.

#### Nonreconstruction accounts

Heycock (2005, 2019) hypothesizes that the low reading of intensional superlatives is not due to their reconstruction within the relative clause, but to the possible lowering of the negation that their interpretation induces.

(23)
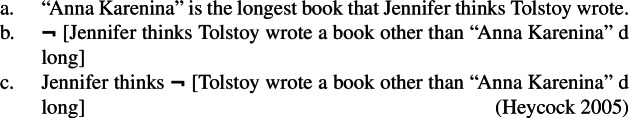

Due to the meaning of the superlative *longest*, sentence (23)a is assumed to generate the negative entailment in (23)b (cf. Giannakidou 1997), which is compatible with the high reading. Assuming a semantic analysis of neg-raising, Heycock further hypothesizes that due to the neg-raising property of *think*, the negation can be interpreted lower as in (23)c, thus triggering the low reading.

According to Heycock, this hypothesis is supported by the following generalization: all and only neg-raising predicates support the low reading. The low reading is blocked by intervening elements such as negation or negative verbs (see (22)), adverbs like (high) *ever* or *mistakenly* (see (24)a), or various predicates including implicatives like *manage*, weak and strong (vs. midscalar) deontic or epistemic operators like *need* or *be possible*, or factives like *know* (see (24)b), which block neg-raising.

(24)
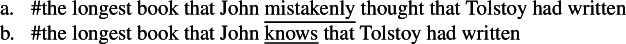

Such intervention effects, however, are unexplained under a reconstruction account. Furthermore, Heycock’s account can straightforwardly explain the NPI facts as well as the specificity of superlatives as compared to other modifiers.

That said, Heycock’s neg-raising approach also faces outstanding problems. First, Bhatt and Sharvit (2005) show that the generalization motivating Heycock’s neg-raising account is in fact incorrect. On the one hand, Heycock’s hypothesis undergenerates because the low reading is available with some predicates blocking neg-raising like *say* (see (8)), *agree*, *be certain* or *hope*, as illustrated in (25).

(25) The longest book John hopes he will (ever) have to read is “Anna Karenina”. (Bhatt and Sharvit 2005)

On the other hand, Heycock’s account overgenerates because the low reading is unavailable with some neg-raising predicates such as *be likely* or *should*.

(26) # The tallest man Mary {is likely to/should} meet is John. (Bhatt and Sharvit 2005)

Second, Heycock’s underspecified account cannot precisely derive the low reading. Even if we specify it using Heim’s (2000) semantics of neg-raising verb (cf. Hulsey and Sauerland 2006, Heycock 2019) and Heim’s (1995/1999) semantics of superlatives (cf. Bhatt and Sharvit 2005), it remains unclear how *d-long* can be interpreted in the scope of the intensional predicate in (23) (this issue is not discussed in Heycock’s articles focusing on *only*). As shown in (27), Sharvit (2003) does spell out a derivation with split scope of three-place -*est* (see (28)) and *d-long* (cf. Hulsey and Sauerland 2006: (53)d).

(27) the est-C 2 1 [John said-w 3 Tolstoy had written-w_3_ (the) d_2_-long-w_3_ book_1_] (Sharvit 2003)

(28)
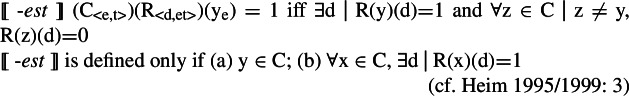

But this analysis requires a stipulation about the domain of comparison to avoid overgeneration. (27) implies reference to the book satisfying the following conditions: there is a degree such that according to John, the book is long to that degree and Tolstoy wrote it; and for all alternative books, it is not the case that according to John, they are long to that degree and Tolstoy wrote them. This derives the correct interpretation only if the set of alternative books is restricted to books that John said Tolstoy wrote.

Overall, no existing account can thus straightforwardly derive the correct interpretation for the low reading. Furthermore, each account fails to derive at least a subset of the properties exhibited by intensional superlatives.

### The superlative clause hypothesis: A novel solution to the problem

The superlative clause hypothesis, I propose, can solve these problems by exploiting ideas of both types of accounts. The core idea is to treat the purported relative clause under low (and some so-called high) readings as a superlative clause, that is, as a degree clause complementing the superlative morpheme and denoting the domain of comparison, as shown in (29)a. This construction is the counterpart of the comparative construction in (29)b.

(29)
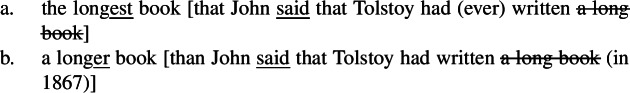

As detailed below, this hypothesis straightforwardly incorporates the improvements of the neg-raising account on the reconstruction account. Since it crucially entails that the superlative morpheme -*est* remains external to the clause, it makes the same correct predictions regarding intervention effects for NPI. Since it is intrinsically tied to the presence of a superlative, it avoids the overgeneration of low readings for other modifiers than superlatives. Furthermore, it overcomes the problems faced by the neg-raising account. The nature of the superlative clause (a degree clause) suggests a natural explanation for intervention effects that does not suffer over- and undergeneration. Moreover, this proposal motivates the need for split scope to derive the correct interpretation without requiring any additional stipulation regarding the domain of comparison, which is explicitly expressed by the clause.

In the next sections, we review in turn how each issue is treated under the superlative clause hypothesis as previewed in (30) (see also Table 1).

(30)

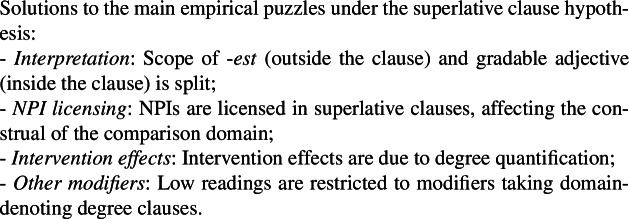

#### Interpretation

We saw that the interpretation of intensional superlatives under the low reading gives rise to a conundrum: on the one hand, the superlative (e.g., *longest*) seems to be interpreted in the scope of the intensional predicate (e.g., *said*); on the other hand, interpreting the superlative morpheme -*est* within the embedded clause generates semantic problems. As suggested by Sharvit (2003) and implied by Heycock’s (2005) analysis, split scope solves the problem: while the gradable predicate is interpreted within the clause, -*est* is only interpreted outside the clause. But both split scope and comparison domain restriction have to be stipulated under previous accounts. By contrast, the superlative clause hypothesis naturally motivates both points. First, the superlative clause hypothesis does not require stipulating a restriction of the domain of comparison to avoid overgeneration: the core feature of this hypothesis is that the clause explicitly expresses the domain of comparison C, which is left implicit in previous analyses (whether it is determined by context with three-place -*est* in (28) or by focus with two-place -*est* in (16)). In (29)a, repeated below, the comparison can thus only concern books by Tolstoy according to John, since the clause explicitly restricts the domain of comparison to those.

(31)

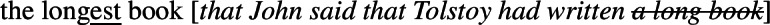

Second, the superlative morpheme -*est* must be interpreted external to the clause since the clause in (32)a is construed as an argument of the superlative morpheme -*est*, just as in (32)b, the comparative clause is an argument of the comparative morpheme -*er*. Note that (32)b shows a simplified representation of the comparative clause under the standard hypothesis that comparative clauses involve abstraction over degrees due to covert operator movement and binding of the degree variable d (see Lechner and Corver 2017; Lechner 2020, i.a.).

(32)
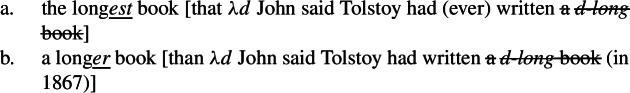

As for the gradable predicate *d-long book*, it must be interpreted both in the embedded clause and in the matrix clause as is the case in comparative clauses (however this is derived^5^). The superlative clause hypothesis therefore entails split scope of -*est* and *d-long*, thus overcoming the aforementioned problems of scoping -*est* within the clause.

More specifically, we mentioned in the introduction that most analyses of comparative clauses agree on treating them as degree clauses complementing a two-place degree-based comparative morpheme. Our hypothesis can thus be implemented by treating clausal superlatives in a parallel fashion by adopting Heim’s (1995/1999) treatment of -*est* in (16) (repeated below in (33)) as a two-place degree-based superlative morpheme, as proposed by Howard (2014) and Romero (2013) – the only two previous proponents of the superlative clause hypothesis.

(33)
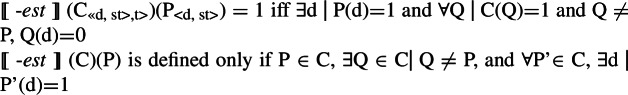

Under this hypothesis, the superlative clause explicitly expresses the domain of comparison C (a set of degree properties^6^), which was originally conceived under this hypothesis as corresponding to the implicit focus value of P. This is illustrated in (34) based on Howard’s 2014 example and representations.

(34)
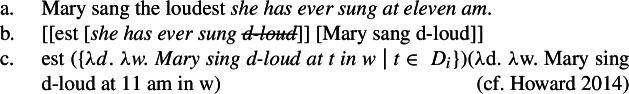

As we can see in (34)c, the superlative clause here corresponds to the first argument of -*est* (after movement of -*est* to a proposition-denoting node) and denotes the set of degree properties – varying along the dimension of singing times – such that Mary sung to those degrees. It is similar to the comparative clause in (35).

(35)

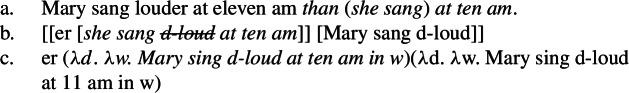

As mentioned in the introduction, superlatives nevertheless differ from comparatives in partitivity: the domain of comparison must contain the correlate. While the comparative clause denotes a set of degrees (under Heimian approaches), the superlative clause thus denotes a set of such sets (or more specifically, intensions thereof): while -*er* relates two elements, -*est* makes a universal claim (see Heim 1985, 1995/1999). Superlatives thus induce a partitive presupposition (P ∈ C in the definedness condition in (33)). As observed by Howard, this entails that mismatches between the matrix (the correlate) and the superlative clauses are impossible, while this is routinely attested in comparatives, as exemplified in (36).

(36)
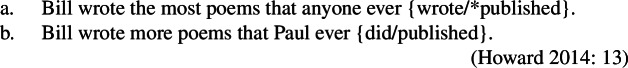

Due to the definition of focus values (e.g., in Rooth 1992), the partitive presupposition is necessarily satisfied under the original hypothesis, where C corresponds to the focus value of P. Under Howard’s hypothesis that C can be explicitly expressed by a superlative clause, this condition entails a match between the matrix clause and the superlative clause.

This matching condition raises a question for applying Howard’s hypothesis to our intensional superlatives, where the matrix clause does not match the subordinate clause. If the subordinate clause corresponds to the domain of comparison under our hypothesis, what exactly then is the correlate in our cases (i.e., the counterpart of the matrix clause in Howard’s cases, e.g., in (34): *Mary sung the loudest at eleven am*, i.e., *λd*. *λw. Mary sing d-loud at 11 am in w*)? It can’t be the overt matrix clause, which need not involve an intensional predicate as in (37):

(37) “Anna Karenina” is the longest book John said Tolstoy wrote.

Under the low reading, we clearly do not want to compare the *actual* length of “Anna Karenina” with the lengths John attributes to Tolstoy’s books: the correlate also needs to be relativized to John’s opinion. Furthermore, doing so would violate the partitive presupposition.

For these reasons, I propose that the correlate is elided, thus matching the superlative clause (given identity conditions on ellipsis) as shown in (38) (cf. Bassi 2021,7).

(38)
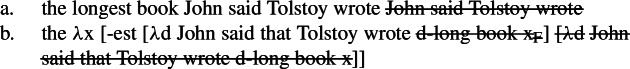

Assuming that the head of the superlative clause reconstructs, (38) thus refers to the book^8^ satisfying the following conditions: there is a degree such that according to John, the book is long to that degree and Tolstoy wrote it; and for all alternative books, it is not the case that according to John, they are long to that degree and Tolstoy wrote them. This derives the correct interpretation.^9^

It remains to specify how the superlative clause can denote a set of degree properties, while the elided clause (the correlate) denotes a degree property as required by the partitive presupposition of superlatives. Assuming like Howard (2014) that focus and NPIs can create sets in similar ways, I hypothesize that set creation in the superlative clause can here derive from focus on the trace x of the head as in (38)b and (39)a (cf. Bhatt 2006) or from the presence of an NPI like *ever* in the superlative clause as in (39)b (cf. Howard 2014, see further discussion in Sect. 2.2.2).

(39)
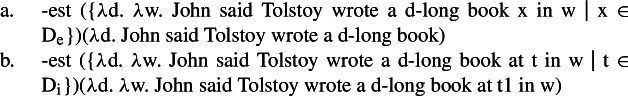

(39)a thus induces quantification over individuals and (39)b quantification over times in the domain of comparison denoted by the superlative clause. The correlate expressed by the elided clause, however, does not denote a set. In (39)a, this derives from the hypotheses that the trace is not focused in the elided clause (and further note that unlike Bhatt’s proposal, focus association does not overgenerate readings due to constraints on elision under identity: for example, focus on *Tolstoy* cannot here generate a reading involving other authors, as *Tolstoy* must be copied under identity in the elided clause). In (39)b, it suffices to hypothesize that the implicit counterpart of the NPI in the elided clause is not a universal quantifier, but a deictic or an existential quantifier (represented as t1 in (39)b). This is independently observed in (40) where *then* is optionally explicit (and further note that as observed by Merchant 2010, polarity items generally license the ellipsis of their nonpolarity counterparts and vice versa).

(40)
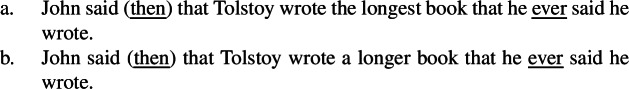

Thus, I hypothesize that the superlative clause contains elements inducing set creation, such as focused elements or NPIs, which can have nonset-inducing counterparts in the elided clause. Note that I do not conversely assume that the elided clause is the superlative clause, and the overt clause the correlate, because we do observe overt NPIs in the overt clauses and it would be difficult to assume focus only in the elided clause (we could, however, in principle assume (implicit) NPIs only in the elided clause; see Sect. 3.2 presenting such cases).

In sum, instead of building the domain of comparison C based on context or focus alone, which we saw overgenerates readings, we have built it in the syntactic representation, as is the case in comparative constructions. Moreover, the ellipsis process we hypothesize, which we independently know is subject to identity conditions, guarantees that the partitive presupposition of superlatives is satisfied: unlike comparatives that relate two distinct elements, superlatives relate a set and an element belonging to that set.

As a final note on the interpretation of intensional superlatives, notice that this proposal bears some resemblance to one of the hypotheses discussed by Hulsey and Sauerland (2006: 128) and Sharvit (2003, 2007), who consider a LF for intensional superlatives that involves split scope of (three-place) -*est* and gradable predicates, although they do not treat these cases as comparable to degree clauses in comparative constructions (under their hypotheses, these clauses remain relative clauses with partial reconstruction and the domain of comparison is contextually determined).

(41)
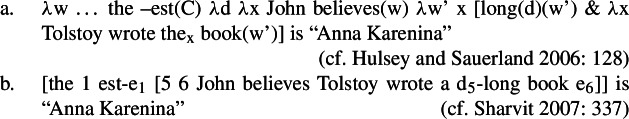

Both Hulsey and Sauerland (2006) and Sharvit (2007) discuss a potential problem for such LFs that seems to extend to our proposal. Consider a scenario where John is sure that “Anna Karenina” is 1000 pages but is unsure whether “War & Peace” is 500 pages or 1500 pages (and he is sure that all other Tolstoy’s books are shorter). In this case, (41) is wrongly predicted to be true because there are some worlds compatible with John’s beliefs where “War and Peace” is not 1000 pages long while “Anna Karenina” is 1000 pages long in all these worlds. But as observed by Hulsey and Sauerland (2006), this problem is solved if we treat the intensional verb as including a uniformity presupposition as proposed by Heim (2000):

(42) 〚believe〛 (w)(P)(x) is defined only if ∀w’∈ Dox(x,w): P(w’) = 1 or ∀w’∈Dox(x,w): P(w’)=0

This implies that when we assert that x believes P, we presuppose that x’s beliefs are determinate, i.e., x either believes P or believes not-p. Therefore, *John believes that* “Anna Karenina” *is d-long* is undefined for degrees between 500 and 1500, and (41) is correctly predicted to be a presupposition failure in the aforementioned scenario.

Now, this kind of uniformity presupposition is usually assumed to be specific to neg-raising verbs and as discussed in Sect. 2.1.2, the low reading is also available with some nonneg-raising verbs such as *say* or *agree*. This leads Sharvit (2007) (unlike Sharvit 2003)^10^ to reject this LF. But although the verb *say* is syntactically not neg-raising, the semantics of *say* is less understood than the semantics of *believe* as mentioned by Hulsey and Sauerland (2006); in fact, it seems reasonable to assume that verbs like *say* or *agree* include the verb *believe* in their semantics (roughly, *say* = *believe* + *utter*) when they take sentient subjects (cf. Anand et al. 2017, Demirok et al. 2019, Major and Stockwell 2021, i.a.).^11^ Under this assumption, the same presupposition failures ensue with those verbs under similar scenarios (i.e., if John says: ````Anna Karenina” is 1000 pages but I don’t know whether “War & Peace” is 500 pages or 1500 pages”, I cannot truthfully report this as “Anna Karenina” *is the longest book John said Tolstoy wrote*).

Furthermore, Sharvit (2007) mentions a second problem related to, but different from the problem just discussed: (41) is perfectly felicitous even if John does not have any opinion about the specific lengths of Tolstoy’s books but only has a comparative belief, i.e., if he believes the following: ````Anna Karenina” is the longest book Tolstoy wrote but I have no idea how long it is.”

Again, the problem can be solved by assuming a uniformity presupposition for intensional predicates. Under the above scenario and our proposal, John’s beliefs are expressed as in (43)a and (41) as in (43)b.

(43)
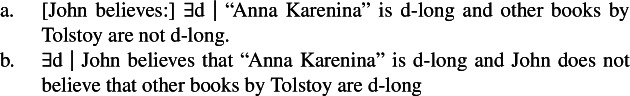

Given the uniformity presupposition of *believe*, (43)a and (43)b are equivalent and our proposal thus correctly predicts (41) to be true under the above scenario. Assuming a uniformity presupposition for nonneg-raising verbs like *say* or *agree* that incorporate the notion of belief can similarly solve the problem. Alternatively, note that Sharvit (2007: fn. 10) suggests that this extensionality problem can be solved by adopting an intensionalized version of -*est*.^12^

#### NPI licensing

The previous section briefly introduced in (39)b how NPIs can play a crucial role in the construal of superlative clauses. In this section, we more specifically show that the superlative clause hypothesis correctly derives the properties of NPI licensing with intensional superlatives as long as the relevant readings are reexamined and the meaning contribution of *ever* is carefully taken into account.

Recall from Sect. 2.1.1 that according to Bhatt, the correlation between the position of *ever* and the type of reading supports a reconstruction account (see (12)): high *ever* in (44)a is only compatible with the high reading because *longest* cannot license high *ever* from its reconstructed position; low *ever* in (44)b is only compatible with the low reading because as wrongly assumed by Bhatt, it must be locally licensed (see Bhatt and Sharvit 2005 for an alternative reconstruction account).

(44)
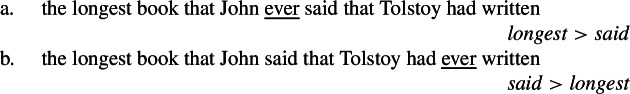

Under Heycock’s nonreconstruction approach, low *ever* is, however, compatible with either reading in the presence of a neg-raising predicate and only with the high reading with a nonneg-raising predicate (since superlatives are NPI licensors). And it is because it blocks neg-raising that high *ever* obligatorily triggers the high reading.

As detailed below, the split scope implied by the superlative clause hypothesis provides a way to resolve this empirical and analytical disagreement: the low reading (in the sense of scoping *d-long* – vs. *longest* – low), and crucially only this reading, is in fact compatible with both low and high *ever*, but the meaning contribution of *ever* gives the illusion that high *ever* triggers a high reading (in the sense of scoping the whole superlative *longest* high).

(45)

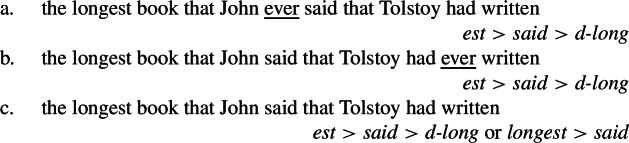

Comparative clauses are known to license NPIs (von Stechow 1984; Heim 1985, i.a.).

(46)
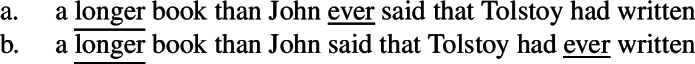

Under the superlative clause hypothesis, it is thus predicted that just as in (46), *ever* can be licensed both in high and low positions when the clause is construed as a superlative clause.

Note that as mentioned in the introduction, NPI licensing is the argument motivating Howard’s (2014) hypothesis that some apparent relative clauses are in fact superlative degree clauses. Howard shows that standard theories of NPI licensing based on Strawson–Downward entailment can predict that superlatives license NPIs only under absolute readings (cf. von Fintel 1999; Herdan and Sharvit 2006; Gajewski 2010); under relative readings, which imply VP scope of -*est*, Strawson downward entailing inferences are invalidated as illustrated in (47).

(47)
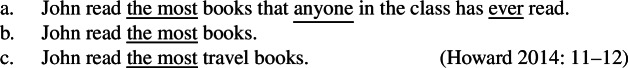

The superlative *the most*, which only triggers relative readings and must thus take VP scope under a Heimian hypothesis, licenses the NPIs *ever* or *anyone*, as shown in (47)a. Yet, (47)b does not Strawson entail (47)c: just because John read more books than anyone else, it does not follow that he read more books of a particular type than anyone else; -*est* does not create a downward entailing environment in its VP complement.

As Howard argues (cf. Bumford and Sharvit 2022), treating the clause in (47)a as a superlative degree clause solves the conundrum: just like *every*, -*est* is not downward entailing with respect to its scope, but it is with respect to its restrictor; in other words, -*est* creates a downward entailing environment in C, but not in P under Heim’s lexical entry in (33). Under the hypothesis that the clause explicitly denotes C, it follows that it is Strawson downward entailing and thus licenses NPIs. In fact, (48)a does entail (48)b provided that John is a syntactician.

(48)
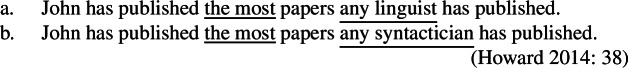

But Howard (cf. Bumford and Sharvit 2022) only applies this explanation to relative readings: under absolute readings, so the argument goes, clauses like the bracketed one in (49) cannot be treated as superlative clauses because under this construal, the correct truth conditions cannot obtain under Heim’s lexical entry in (33), as discussed in Sect. 2.2.1.

(49) “War and Peace” is the longest book [Tolstoy ever wrote]. (Howard 2014: 50)

The point of the present article is instead to argue that such bracketed clauses can in fact also be treated as superlative clauses; and in Sect. 2.2.1, we discussed ways to overcome the semantic problems raised by this construction, which we argue are solved by assuming ellipsis of an identical clause.^13^ In fact, Howard’s theory-independent argument in (48) carries over to (49): (50)a entails (50)b provided that Tolstoy wrote “War and Peace” in the 1860s.

(50)
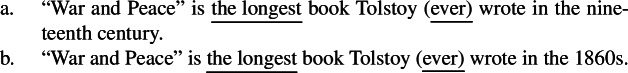

In sum, our superlative clause hypothesis predicts that NPIs like *ever* can be licensed in any position in the clause construed as a superlative clause.

Given that under our hypothesis, we saw that the so-called low reading derives from the superlative clause construal entailing split scope, it should thus be compatible with both *low* and high *ever*. But this seems to go against Bhatt and Heycock’s converging claim that high *ever* only triggers the high reading. This apparent problem, I claim, is resolved by the split scope hypothesis implying that high *ever* triggers a low reading – in the sense that book lengths are evaluated by John – that resembles the so-called high reading – in the sense that the superlative comparison is done by the speaker.

More specifically, the NPI *ever*, which quantifies over time, affects the interpretation of the comparison set under our hypothesis, since it is part of the superlative clause as represented in (51).

(51)
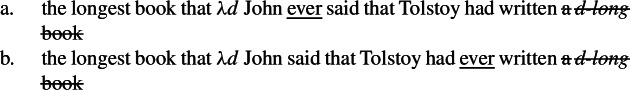

Recall from our discussion in Sect. 2.2.1 that NPI indefinites can crucially contribute to the creation of the set denoted by superlative clauses. For example, *ever* can contribute to creating the comparison set C through quantification over times, as exemplified in (52).

(52)
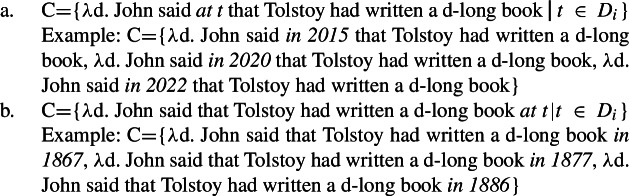

High *ever* in (51)a thus induces the comparison set in (52)a, where the degree sets forming the set denoted by the superlative clause vary along the dimension of John’s saying times: the comparison set includes lengths of books by Tolstoy that were mentioned by John at different times (e.g., in 2015, 2020, 2022). Low *ever* in (51)b, however, induces the comparison set in (52)b, where the degree sets forming the comparison set vary along the dimension of Tolstoy’s writing times according to John: the comparison set includes lengths of books that were written by Tolstoy at different times according to John (e.g., in 1867, 1877, 1886).

This distinction gives rise to a difference of interpretation that resembles that invoked for distinguishing between the low and the high readings, although both interpretations correspond to variants of the low reading under my hypothesis (in the sense that *d-long* is interpreted low). One way used by Bhatt (2002) to paraphrase the low vs. high readings in the case of *first* is to specify whether it is the order of saying or the order of writing that matters. In the case of *longest*, the difference focuses on whether the comparison is made between lengths of books mentioned at different times ((52)a) or written at different times ((52)b). In both cases, the comparison is explicitly expressed to be made by the speaker (-*est* scopes over *said*). In (52)b, it is implied that John also made the comparison at least implicitly, since he expressed an opinion about all relevant book lengths (presumably at the same time in the absence of indication to the contrary), under the assumption that his thinking obeys logical rules. But in (52)a where John expressed opinions about book lengths at different times, this implication does not necessarily hold: making a length comparison requires not only knowing the lengths of the elements to be compared and the logical ordering rule, but also holding all lengths simultaneously in memory. This consideration explains how the other common paraphrase used for the high vs. low readings, namely “longest according to the speaker vs. John” can correspond to our two variants of the low reading.

Our hypothesis thus implies that the so-called high reading corresponds to two possible logical forms, and some confusion in the literature comes from the near equivalence of these LFs under some circumstances, which can be described using various (potentially misleading) paraphrases.

(53)
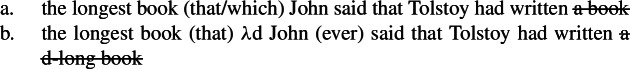

Specifically, the first LF (that assumed by all the previous literature for the high reading) involves high scope of the whole superlative. Under my hypothesis, this is the LF in (53)a under which the clause is interpreted as a standard relative clause (vs. a superlative clause); for a subset of speakers, the use of *which* (vs. *that*) relativizers forces this construal (see discussion below around (73)). Under this LF, John need not be opinionated about book lengths and the comparison is done by the speaker among books by Tolstoy according to John. The second LF is the same LF I assume for the low reading, namely the one in (53)b where the clause is construed as a degree superlative clause and *d-long* (vs. -*est*) is interpreted low. Under this LF, John is opinionated about book lengths and the speaker is responsible for making the comparison between these lengths assumed by John; by default, John’s opinion about book lengths implies a comparative judgment by John (which thus amounts to Bhatt’s low reading), but this is not necessarily the case if these opinions are spread over times as is forced by the modification of the intensional predicate by *ever* (which thus amounts to Bhatt’s high reading). In sum, both low and high *ever* can be associated with a superlative clause construal, and the difference of reading does not derive from a scopal difference of (part of) the superlative adjective, but from the difference of interpretation of the comparison class induced by the placement of *ever*.^14^

This hypothesis is further supported by Heycock’s (2019) observation that the placement of *ever* does not correlate with binding Condition A, as shown in (54).

(54) That is the first picture of himself_i_ that I ever thought Freud_i_ might sell. (Heycock 2019: 96)

Contrary to Bhatt’s predictions and in support of ours, *himself* can be bound in the low clause by *Freud* (which is not construed as a logophoric center here and cannot thus yield exemption from Condition A) in (54) involving high *ever*.^15^

Yet further corroboration of our hypothesis comes from the fact that intervention effects with negative verbs (see Sect. 2.1 and further discussion in Sect. 2.2.3) are not just observed with low *ever*, but also with high *ever*:

(55)
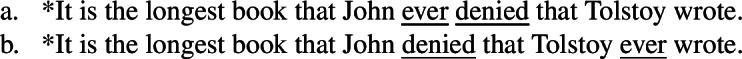

Conversely, subject nonmodal infinitive clauses (which denote the domain of comparison as will be discussed in Sect. 2.2.4) do not only license low *ever*, but also high *ever*:

(56)
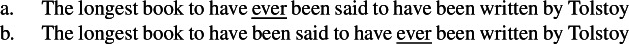

Moreover, nonreferential readings (see below discussion in (71)) are available with high *ever*:

(57) The longest book John will ever need to read to pass his classes should not exceed 1000 pages.

To complete the argument, we must finally clarify how NPI licensing works under relative clause construals. So far, we have explained why both high and low *ever* are compatible with a superlative clause construal, and why under this construal, high *ever* can be described as entailing a high reading, and low *ever* a low reading. But our hypothesis does not exclude a relative clause construal, which induces a high reading. How does it interact with NPI licensing?

Given the monotonicity profile of two-place -*est* we discussed above, our hypothesis implies that NPIs can only be licensed if they occur in the domain of comparison (i.e., in C). If the clause is construed as a relative clause outside the domain of comparison, it is correctly predicted not to license NPIs. This reading can be facilitated if we add another possible explicit domain of comparison, as in (58).

(58)
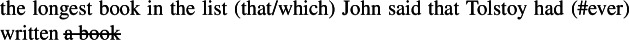

If the comparison is established among books on the list (of reading assignments, for example), the clause must be construed as a relative clause whose semantic contribution is to restrict the reference of the longest book of the list to be a book by Tolstoy according to John; under that reading, *ever* is unacceptable.^16^

The last issue bears on whether relative clauses can be construed as individual-based domains of comparison, under the assumption that there are two possible superlative morphemes, i.e., one two-place degree-based and one three-place individual-based (as is debated in the literature on comparatives). If so, such relative clauses are predicted to license NPIs. Note that this is also von Fintel’s 1999 prediction since under his hypothesis, NPIs can be licensed under absolute readings as long as they appear in the NP argument of the superlative (see fn. 14). Furthermore, this hypothesis does not impose any restriction on the position of NPIs within the clause, so that both low and high *ever* are predicted to be acceptable in such clauses. Low *ever* is therefore predicted to be compatible with a high reading (i.e., *ever* should be able to modify *wrote* in (51)b even when *d-long* is not interpreted low). This means that (51)b should be expected to refer to Tolstoy’s longest book according to the speaker in a scenario where the speaker and John disagree about the lengths (especially the highest one) of Tolstoy’s books (but not about authorship). This is precisely what is claimed not to be the case by the previous literature: low *ever* forces the ascription of the length judgment to John (vs. the speaker). This judgment seems to be supported by the contrast between (59)a and (59)b: the high reading with low *ever* seems clearly less available in (59)b than in (59)a when the relative clause appears in a partitive construction (and is thus forced to be both individual-based and within the domain of comparison).

(59)
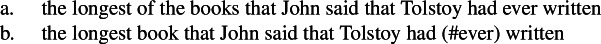

This observation suggests that relative clauses cannot be construed as individual-based domains of comparison. Why this would be so remains to be further investigated.^17^

#### Intervention effects

As discussed in Sect. 2.1.2, intensional superlatives are subject to intervention effects, and Heycock (2005) takes the interveners to be nonneg-raising predicates; but Bhatt and Sharvit (2005) argue that this hypothesis both over- and undergenerates. Instead, the superlative clause hypothesis directly accounts for the noncontroversial cases of intervention effects such as negation, and provides an explanation as to why some cases remain empirically debated. Under our hypothesis, intervention effects are reduced to those observed with degree quantification: since the superlative clause is treated as a degree clause, it is predicted to be subject to the same intervention effects as degree questions or comparatives as illustrated in (60) with negation.

(60)
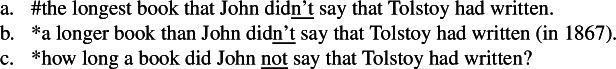

In other words, the superlative clause hypothesis straightforwardly derives the negative island effects that Bhatt (2002) mentions without being able to explain. It’s been noticed since at least Ross (1984) that negative elements interfere with some types of wh-movement. Although both the exact empirical generalization and the analysis remain debated (see Rizzi 1990; Szabolcsi and Zwarts 1993; Rullmann 1995; Abrusán and Spector 2011, i.a.), it is uncontroversial that intervention effects with the negation itself or negative verbs such as *deny* arise both with degree and amount quantification. While intervention effects yield ungrammaticality in the former case as in (61)a, they constrain the interpretation to referential readings in the latter case as in (61)b.

(61)
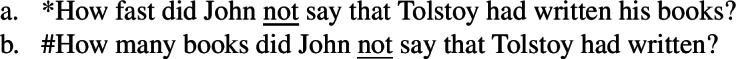

For our purposes, we do not need to take a stand on how to analyze negative islands: it suffices to observe that the same intervention effects arise for the low reading of intensional superlatives and for other well-known cases of negative islands. For example, it has been observed that negative islands can be obviated by some properly placed modals (Fox and Hackl 2007):

(62)
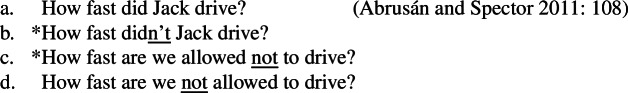

Strikingly, the same holds with our low readings:

(63)

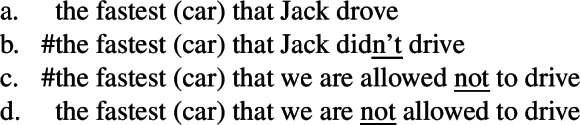

That said, Heycock explicitly points out some cases of intervention that seem to arise with our low readings, but not with amount quantification. Bhatt and Sharvit (2005) also implicitly mention such cases when they argue against Heycock’s generalization that *should* or *be likely* do not trigger low readings (such predicates do not give rise to negative islands). Such intervention effects, I argue, are artefacts of the way Bhatt (& Sharvit) and Heycock describe the low readings.

First, note that Heycock makes her point using *only* and *first* (vs. run-of-the-mill superlatives like *longest*). As mentioned by Bhatt & Sharvit, this is problematic in cases in which the high reading includes the low reading, which often happens with *only*. Furthermore, note that the paraphrases used by both Bhatt and Heycock for *only* and *first* usually amount to interpreting the whole modifier within the clause, which is arguably not the correct way to derive the low readings. In the absence of a fully spelled out analysis for *first* and *only* (see suggestions in Appendix, Sect. A.1), it thus seems safer at this point to reason on intervention effects based on standard superlatives like *longest* (as also argued by Bhatt and Sharvit 2005).

Bhatt, Sharvit, and Heycock’s reasoning about them is confounded, I argue, by their never considering nonreferential readings. For lack of space, I illustrate this point using only one example that both Bhatt & Sharvit and Heycock treat as a case of intervention effect (although they explain it differently); but as far as I can see, the point extends to all other cases. Specifically, Bhatt and Sharvit (2005: 74) and Heycock (2005: 371) claim that the strong deontic operator *need* intervenes for the low reading.

(64)

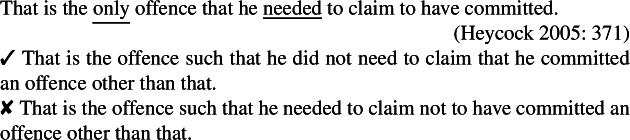

(65)
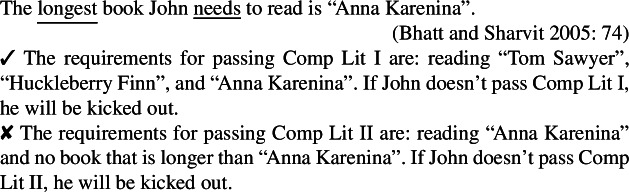

Heycock claims that the meaning indicated by the second paraphrase in (64) is unavailable because *need* blocks neg-raising, and Bhatt & Sharvit claim that the second scenario in (65) is inappropriate because the low reading here entails the high reading. But both (64) and (65) crucially exhibit a referential reading of *the only*/*longest book*, unlike corresponding low readings of amount or degree questions (to which both Bhatt and Heycock compare low readings); under their low reading, both (66) and (67) only require numbers (of offences or pages, respectively) as answer, not specific entities (offences or books).

(66) How many offences did he need to claim to have committed (to be credible)?

(67) How long a book does John need to read (to pass Comp Lit I)?

The same holds of degree or amount comparatives:

(68) I committed more offences than he needed to claim to have committed (to be credible).

(69) I read a longer book than John needs to read (to pass Comp Lit I).

Crucially, the low reading becomes similarly available if we modify (64) and (65) accordingly:

(70) The only offence that John needed to claim to have committed needed not be important.

(71) The longest book John needs to read to pass Comp Lit I need not be in a foreign language.

Because they do not involve an identificational construction (e.g., *that is X*, *X is* “Anna Karenina”), (70) and (71) are compatible with a nonreferential reading that highlights the low reading. Recall that under our hypothesis, only *d-long* (not -*est*) is interpreted in the scope of the intensional predicate under the low reading. Accordingly, (71) is felicitous in scenarios in which only a certain length of book (vs. a specific book) defines the requirements, e.g., if to pass Comp Lit I, John must read two specific French and German 50-page novels as well as any 500-page novel (e.g., taken from a list); in those cases, the book in question is not specific (unlike in (65) where the longest book is specific although the shorter books are not). Similarly, (70) favors an interpretation where no specific offence is in question: the crucial point is that John had to claim to have committed only *one* offence (vs. only *that* offence in (64)).

The hypothesis that the intervention effects for our low readings can be reduced to intervention effects for degree quantification is further supported by the behavior of intensional superlatives in the presence of *which* relativizers, which parallels the behavior of so-called amount relatives like (72).

(72) It will take us the rest of our lives to drink the champagne {that/ % which} they spilled at the party. (cf. Heim 1987: 38)

(73) the longest book {that /% which} John said that Tolstoy had written.

Amount relatives, which are standardly argued to involve degree relativization (see Carlson 1977; Heim 1987, Grosu and Landman 1998, Herdan 2008, i.a.) are claimed to disallow *wh*-relativizers (at least for a significant portion of speakers). For instance in (72), the amount reading, under which it is the amount of champagne (vs. the actual champagne) spilled that is under discussion, is unavailable with *which* (vs. *that*). Similarly, the low reading is absent (at least for a large number of speakers) in (73) when it involves a *which*-clause (cf. Howard 2014: fn.7 and fn.17).

In sum, the superlative clause hypothesis provides a straightforward solution to the problem of intervention effects: because it involves degree relativization, it predicts negative islands effects (and any other intervention effect observed with degree quantification); other purported intervention effects are illusory and due to the interpretive constraints derived from split scope.

#### Other modifiers

Finally, the superlative clause hypothesis straightforwardly solves the problematic point concerning other modifiers. Recall that Heycock (2005) shows that the seemingly low reading of other modifiers like *wonderful* does not exhibit the same properties as the low reading of superlatives, leading her to conclude that they are not derived in the same way. This undermines Bhatt’s reconstruction account and supports her neg-raising account according to which only modifiers generating a negative entailment are predicted to give rise to low readings.

The predictions of the superlative clause hypothesis are similar to Heycock’s hypothesis: it correctly predicts that only a specific class of modifiers can trigger the low reading (associated with neutral intonation, obligatory intensional predicate, and intervention effects). Specifically, only modifiers compatible with superlative clauses are predicted to give rise to the low reading. This straightforwardly makes the correct predictions for our main cases involving superlatives like *longest*.

What about the other cases discussed by Bhatt (2002) and Heycock (2005)? Even if they disagree on the analysis and on some empirical details, Bhatt (2002) and Heycock (2005) agree on including in the descriptive class of intensional superlatives not only superlatives like *longest*, but also adjectival *only*, ordinals like *first* and numeral-like modifiers like *few*, and on excluding (evaluative) adjectives.

(74)
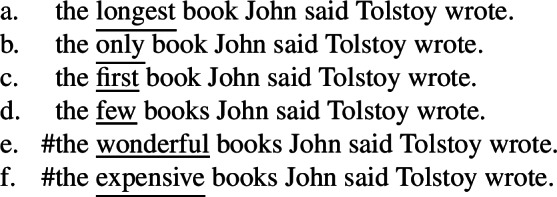

Our superlative clause hypothesis suggests a solution as to the relevant class of modifiers that can trigger the low reading: superlatives, *only*, ordinals and numerals are all compatible with a degree-based domain argument or comparison class, which, I hypothesize, can be explicitly expressed by the clause containing the intensional predicate. This is evidenced by the striking fact in (75) that they all (and only they) can take nonmodal subject infinitival clauses (cf. Bhatt 2006), which have been argued to denote comparison classes (Bylinina et al. 2015). All elements are also compatible with *of*-partitives as in (76).

(75)

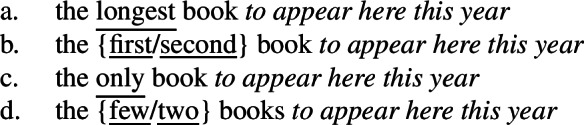

(76)
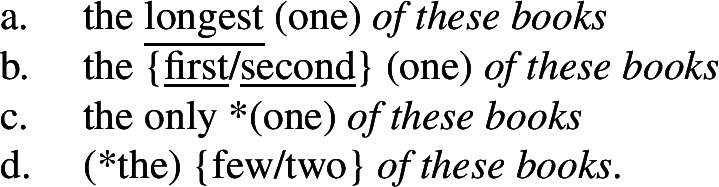

I would thus like to propose that all and only modifiers triggering the low reading are modifiers that can take a domain argument, and the low reading arises when the clause is construed as a degree-based comparison class. A detailed analysis of ordinals, *only* and numerals will have to await further research, but a sketch of how this analysis can apply to each case is presented in the Appendix (Sect. A.1).

In sum, some apparent relative clauses can be construed as superlative clauses, i.e., as degree-based domains of comparison, and this construal can explain all the properties of the so-called low reading of intensional superlatives. The goal of the next section is to show that similarly, the superlative clause hypothesis can parsimoniously derive the properties of upstairs *de dicto* readings – the mirror case of intensional superlatives, which involves scopal interaction between superlatives and intensional predicates in the other direction.

## Upstairs de dicto readings

Upstairs *de dicto* readings (as dubbed by Sharvit and Stateva 2002) have been identified by Heim (1995/1999) as a fifth possible reading of sentences like (77) involving an intensional predicate and a superlative.

(77) John wants to climb the highest mountain.

As observed by Heim (1995/1999), (77) is descriptively multiply ambiguous. First, the superlative induces an ambiguity between an absolute reading and a relative reading, depending on whether the comparison set includes all relevant mountains or all relevant climbers (see discussion of (4) and (17)). Second, the intensional predicate triggers an ambiguity between *de re* and *de dicto* readings depending on who the judgment about the highest mountain is ascribed to – the speaker or John. The combination of these two sources of ambiguities gives rise to four possible readings: absolute *de re* (the mountain that John wants to climb is actually higher than all other (relevant) mountains, i.e., Mount Everest), absolute *de dicto* (John wants to climb a mountain he thinks to be higher than all other (relevant) mountains, e.g., K2 or some imaginary mountain that he thinks is the highest of all mountains), relative *de re* (the mountain that John wants to climb is actually higher than the mountains that all other (relevant) people want to climb, e.g., Mount Sainte-Victoire), relative *de dicto* (John wants to climb a higher mountain than all other (relevant) people, it does not matter which one). These readings can be derived if we standardly assume different scope options for the DP *the highest mountain* (below or above *want*) and different choices for the implicit domain of comparison of -*est*.

But Heim (1995/1999) shows that there is yet another, more problematic reading under which the mountain height seems to be determined *de dicto*, but the relative comparison seems to be made *de re*. This reading is salient in a scenario in which the speaker conducts a survey about various people’s athletic ambitions, which reveals, for instance, that John wants to climb a 6000 m high mountain, Mary wants to climb a 4000 m high mountain, and Bill wants to climb a 1000 m high mountain. This reading is relative because the comparison is made between aspirant climbers (vs. mountains). But it is not a relative *de dicto* reading because John does not have any comparative desire (so that *the highest mountain* cannot scope below *want*), and it is not a relative *de re* reading either because there is no specific mountain that John wants to climb (so that *the highest mountain* cannot scope above *want*).

This observation leads Heim to motivate an analysis (which I will henceforth refer to as the movement theory) under which -*est* moves above *want* (i.e., upstairs) while *d-high mountain* remains below it (i.e., can be read *de dicto*). This is shown in (78) based on two-place -*est* (see (16)) (but the same can obtain using 3-place -*est* in (28), as shown by Heim 1995/1999).

(78)
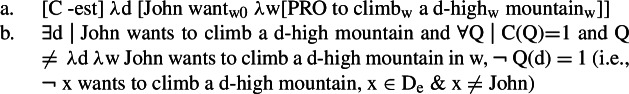

(78) involves covert movement of -*est* to the propositional level. The domain of comparison consists in a set of degree properties corresponding to the focus value of the complement of -*est* (i.e., in case *John* is focused, the set of properties of degrees d such that x – John or any relevant alternative individual – wants to climb a d-high mountain). Due to split scope of -*est* and *d-high mountain*, it is correctly predicted that a specific desire of climbing achievement is attributed to John that does not involve any particular mountain, while the comparison between climbing desires is made by the speaker.

As shown by Heim (1995/1999: 8–9), the derivation of upstairs *de dicto* readings strongly supports this movement theory over the so-called in situ theory under which -*est* remains within the DP. Whether the DP domain argument is assumed to vary with the desire worlds or not, no value can be found that can express the relevant reading under the in situ theory, unless some machinery specific to relative readings is postulated. Farkas and Kiss (2000) propose that the noun (e.g., *mountain*) can be interpreted in relation to a correlate noun (e.g., *John*) and a predicate (e.g., *want to climb*) through some kind of e-type binding. Sharvit and Stateva (2002) propose that the DP (e.g., *the highest mountain*) can be interpreted as a property. These mechanisms mimic split scope without assuming movement.

Strikingly, the movement hypothesis is the mirror image of the superlative clause hypothesis we have discussed in Sect. 2: in the case of intensional superlatives, the superlative surfaces higher than the intensional verb even if the judgment of measure (e.g., book length) can be made *de dicto*; in the case of upstairs *de dicto* readings, the superlative surfaces lower than the intensional verb even if the comparative judgment can be made *de re*. The conundrum can be solved in both cases by splitting the scope of -*est* and the gradable predicate across the intensional predicate. The goal of this section is to focus on upstairs *de dicto* readings and show that the superlative clause hypothesis is a variant of the movement theory in a system licensing clausal complements for superlatives, which has the conceptual advantage of assimilating superlatives with comparatives. We will detail the analysis of upstairs *de dicto* readings under the superlative clause hypothesis in Sect. 3.1 before evaluating how it fares with respect to the standard movement theory and the in situ theory in Sect. 3.2.

### Upstairs de dicto reading under the superlative clause hypothesis

#### Superlative clause ellipsis

The superlative clause hypothesis implies that in (77), the domain of comparison can be expressed by an elided degree clause as in (79)a, paralleling the comparative clauses in (79)b.

(79)
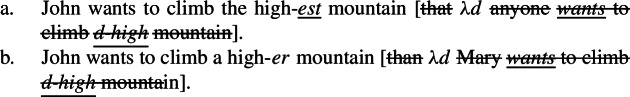

Just as in the case of intensional superlatives, (79)a involves split scope of -*est* and an intensional predicate (*want*). But intensional superlatives and upstairs *de dicto* readings appear as mirror cases of each other because it is the domain of comparison that is here elided under identity with the correlate, while in the case of intensional superlatives, it was conversely the correlate clause that was elided under identity with the domain of comparison.

The ellipsis of a full superlative clause in (79)a can be independently motivated. It is well known that comparative clauses can involve multiple types of ellipsis (see Lechner 2020 for a review). Although the case is hardly studied, it is even possible for the comparative clause to be fully implicit. As discussed in Charnavel (2015) for the case of comparatives and adjectives like *same*/*different*, sentences involving a bare comparative (e.g., *I climbed a higher mountain*) are multiply ambiguous, and this can be explained by assuming different types of elided clauses (or phrases) crucially involving a covert underspecified element that can be interpreted deictically, anaphorically or reflexively (see x below interpretable as *she* in (a), *himself* (*before*) in (b), or *each other* in (c)).^18^

(80)
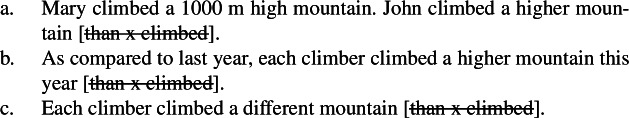

In other words, comparatives can take fully silent clauses as complements when the DP is the standard of comparison (e.g., *Mary* in (79)b), which contrasts with the focused element in the correlate clause (e.g., *John* in (79)b), is a covert underspecified element (i.e., *x* in (80)), and the rest of the sentence is elided under identity.

The superlative clause hypothesis implies that superlatives can involve similar types of ellipsis, including full ellipsis of the clause. As discussed in Sect. 2, the specificity of superlatives as compared to comparatives implies that the standard of comparison must induce computation of a set as the domain of comparison. In (79)a, the silent counterpart of *John* in the domain of comparison must thus correspond to a quantifier (expressible as *anyone*, cf. Howard 2014).^19^ In fact, note that a quantifier can also stand as (overt or covert) standard of comparison in the case of comparatives.

(81) Mary climbed a 1000 m high mountain. Bill climbed a 1500 m high mountain. Lea climbed a 500 m high mountain. John climbed a higher mountain (than anyone).

(82) John climbed 3000 m, 3500 m, and 3800 m high mountains in the past. He climbed a higher mountain (than ever) today!

The uniformity assumed between comparatives and superlatives thus implies that the derivation of upstairs *de dicto* readings relies on ellipsis of a clause like the bracketed one in (79)a, just like that of the equivalent reading in comparatives in (79)b. This hypothesis amounts to unpacking the lexical entry of -*est*, which in Heim’s (2001: 234) terms, involves ‘semantic ellipsis’, and moving some of its ingredients to the syntactic representation. Beyond bringing the lexical entry of -*est* closer to that of -*er*, this hypothesis better motivates movement of -*est* (in parallel to movement of -*er*) due to the interaction it implies between ellipsis and movement, as detailed in the next section.

#### Movement of -*est*

Although – to my knowledge – its relevance to upstairs *de dicto* readings has hardly been exploited, the interaction between comparatives and intensional predicates has been much studied (see Heim 2001; Bhatt and Pancheva 2004, i.a.). In fact, the upstairs *de dicto* reading corresponds to one of the three readings that have been identified in comparative constructions involving intensional predicates such as (83) (see Williams 1974; Sag 1976, Heim 2000, 2001, Bhatt and Pancheva 2004, i.a.).

(83)
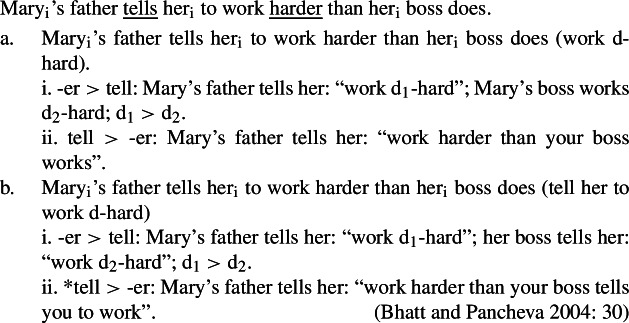

Here, different readings result from different possible choices of ellipsis and covert movement of the degree quantifier, which are partially interrelated. When only the lower VP is elided as in (83)a, the comparative morpheme -*er* can be interpreted above *tell*, in which case the *than*-clause must be read *de re* (see (83)a-i), or below *tell*, in which case the *than*-clause can be read *de dicto* (see (83)a-ii) or *de re* (not illustrated).^20^ When the higher VP is elided as in (83)b, -*er* must scope over *tell* to resolve antecedent containment deletion and license ellipsis (see (84)) so that the *than*-clause must be read *de re* as in (83)b-i (Sag–Williams Ellipsis-Scope generalization). The *de dicto* reading is unavailable in (83)b-ii and can only be triggered by the less elliptical structure in (85).

(84)

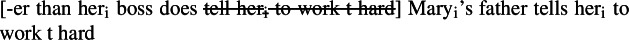

(85) Mary_i_’s father tells her_i_ to work harder than her_i_ boss tells her to.

Crucially for our purposes, the reading in (83)b-i is the comparative counterpart of the upstairs *de dicto* reading: the measure judgment (about how hard Mary should work) is ascribed to the attitude holders (Mary’s father, Mary’s boss), while the comparison is made by the speaker. Similarly, the comparative counterpart of (79)a in (86) (cf. (79)b) exhibits a reading where only the comparison (vs. the measure judgment) is made by the speaker.

(86) John wants to climb a higher mountain than Mary does.

Furthermore, Heim (2001) observes that the reading in (83)a-ii and the reading in (83)b-i extend to superlatives.

(87) Mary_i_’s father tells her_i_ to work (the) hardest.

For example, (87) can either express that Mary’s father’s order is comparative (what Mary is ordered to do is to work harder than others work) or that it is quantitative (what Mary is ordered to do by her father is to work a certain amount; it turns out that this amount exceeds other amounts recommended to her by other people). Heim (2001) takes this observation as further evidence for the possibility of DegP movement above intensional predicates. According to her, the argument is clearer in the case of superlatives because they do not involve syntactic ellipsis and the argument is thus not contingent on assumptions about ellipsis licensing. But the argument can be reversed: the fact that similar readings occur with comparatives and superlatives arguably provides evidence for the hypothesis that they are derived in a similar fashion and that -*est* movement is motivated by ellipsis licensing (antecedent containment deletion). This is our superlative clause hypothesis according to which superlatives do in fact involve syntactic ellipsis as represented in (88) (not indicating -*est* movement).

(88)
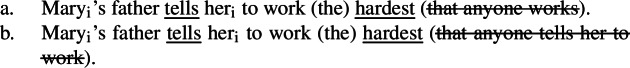

The relative *de dicto* reading is derived by ellipsis of the lower VP as in (88)a (cf. (83)a-ii), while the upstairs *de dicto* reading is derived by ellipsis of the higher VP as in (88)b (cf. (83)b), which requires scoping -*est* above the intensional predicate.

Furthermore, the facts motivating the Sag–Williams Ellipsis-Scope generalization in the case of comparatives are also observed with superlatives. Specifically, (87) does not exhibit the reading in (89), just like we saw that (83) does not display the reading in (85).

(89) Mary_i_’s father tells her_i_ to work (the) hardest that anyone tells her to.

Under Heim’s (1995/1999) analysis, the absence of this reading derives from the hypothesis that the type of comparison depends on the shape of the sister of -*est*.

(90) Mary_i_’s father tells her_i_ [C-est] *λ*d[PRO to work d-hard].

In (90) based on two-place -*est*, it is the case because C corresponds to the focus value of the sister of -*est*: if the degree comparison is *de dicto* as in (89), -*est* must remain below the intensional predicate, which implies that the domain of comparison cannot involve the intensional predicate.

Under our superlative hypothesis, the absence of the same reading as (89) in (87) derives from the same constraints on ellipsis licensing discussed for comparatives.

(91)
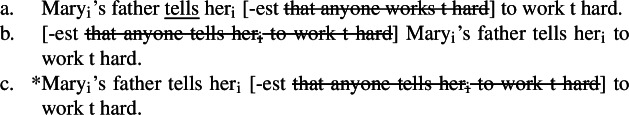

When the higher VP is elided as in (91)b and c, only a degree *de re* reading obtains (i.e., the upstairs *de dicto* reading in (91)b) because -*est* must scope above *tell* to resolve antecedent containment deletion. The degree *de dicto* reading is only possible with lower VP ellipsis as in (91)a.

What about the reading in (83)a-i? While Heim (2001) only discusses two readings with comparatives (the degree *de re* reading with higher VP ellipsis as in (83)b-i, and the degree *de dicto* reading with lower VP ellipsis as in (83)a-ii), Bhatt and Pancheva (2004) mention as a third reading the degree *de re* reading with lower VP ellipsis, which can obtain if -*er* scopes over the intensional predicate as noted in (83)a-i. This reading is discussed based on (92) in Gawron (1995) that observes a difference between comparatives and superlatives.

(92)
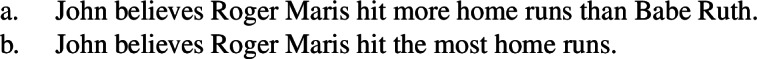

Unlike (92)b, (92)a can be true if John only has a belief about the number of home runs Roger Maris hit, and does not know anything about any other baseball players. In other words, it is only in the case of comparatives (vs. superlatives) that the speaker can be responsible for the comparison between the number of home runs John believes Roger Maris hit with the actual number of home runs other baseball players hit.

Similarly, it seems that (87) lacks the degree *de re* reading with lower ellipsis, i.e., (87) is not true if Mary’s father orders Mary to work a certain amount, but does not know anything about the amount of work ordered or achieved by others, and only the speaker judges this amount as higher than all other relevant amounts of work achieved.^21^

The absence of this reading is derived by the movement theory. As we saw above in (90), this theory predicts a strict correlation between the scope of -*est* and the type of comparison that can be made. If -*est* moves above the intensional predicate, the domain of comparison must thus involve the intensional predicate as shown in (93) using two-place -*est*.

C-est *λ*d[Mary_i_’s father tells her_i_ to work d-hard].

Our superlative clause hypothesis makes the same prediction: the LF in (94) cannot satisfy the partitive definedness condition of -*est* because P (*λ*d. *λ*w. Mary’s father tells her to work d-hard in w) does not belong to the intended C ({*λ*d. *λ*w. x works d-hard in w | x ∈ D_e_}). In other words, the matching condition between the correlate clause and the domain of comparison is not satisfied (see Sect. 2.2.1).

(94)

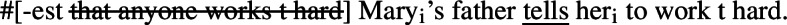

To sum up, our hypothesis correctly predicts that the behavior of superlatives is similar to that of comparatives modulo the partitivity condition that imposes more constraints on superlatives: in principle, it implies the same possibilities on ellipsis licensing in comparatives and superlatives, but superlatives are further restricted by the required match between the correlate clause and the domain of comparison. As now represented with our original example in (95), superlatives thus display a high relative upstairs *de dicto* reading in (a) (corresponding to the high scope reading of comparatives with high ellipsis) and a low relative downstairs *de dicto* reading in (c) (corresponding to the low scope reading of comparatives with low ellipsis), but can trigger neither a low relative upstairs *de dicto* reading in (b) (corresponding to the high scope reading of comparatives with low ellipsis) due to the partitivity condition, nor a high relative downstairs *de dicto* reading in (d) (corresponding to the low scope reading of comparatives with high ellipsis) due to the Sag–Williams Ellipsis-Scope generalization; this last reading only obtains when the intensional predicate is not elided, as in (96).

(95)
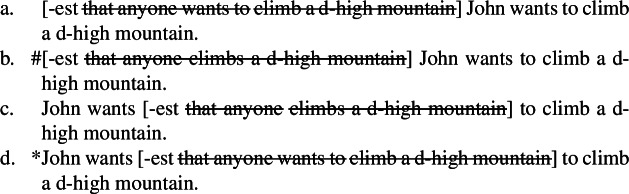

(96) John wants to climb the highest mountain that anyone wants to climb.

Now recall that we originally identified five readings in sentences involving a superlative and an intensional predicate; not just the upstairs *de dicto* and relative *de dicto* readings as in (95)a and (95)c above, but also relative *de re*, absolute *de dicto*, and absolute *de re* readings. We originally saw in (77) that these different readings could be assumed to derive from different scopes of the superlative DP (e.g., *the highest mountain*) with respect to the intensional predicate (e.g., above or below *want*) and different choices of comparison sets (e.g., all relevant mountains vs. all relevant aspirant climbers). Absolute readings also exist in comparatives, as well as the relevant distinction between *de re* and *de dicto* readings, but this requires the comparative adjective to be attributive as in (97): if the intended complement of the comparative is a phrase denoting a mountain (e.g., *Mont Blanc*), the equivalents of both absolute *de re* and *de dicto*, as well as relative *de re* readings, are available.

(97) John wants to climb a higher mountain (than Mont Blanc/than Mary does).

Note that under two-place -*er* and -*est* hypotheses, absolute readings can be captured in a similar way to relative readings by scoping -*er* or -*est* within the NP (cf. Romero 2011) and assuming ellipsis of reduced predicative clauses (cf. Charnavel 2015: 160).

(98)

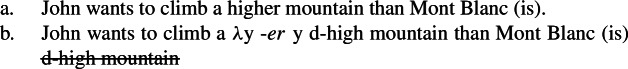

(99)
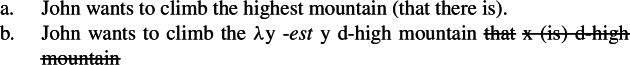

Further note that in parallel to the case in (94), the matching condition of superlatives precludes two further readings that are exhibited by the comparative (i.e., absolute upstairs *de dicto* and low relative upstairs *de re*). First, (98)a can express a comparison between the climbing ambitions of John (in terms of height, but not targeting any specific mountain) and the actual height of Mont Blanc; (99)a does not have the corresponding reading (or only marginally, see fn. 22), because the superlative clause must match the correlate to satisfy the partitive condition. Similarly, *John wants to climb a higher mountain than Mary* *did* can express a comparison between the climbing ambitions of John in terms of a specific mountain and the actual climbing achievement of Mary; *John wants to climb the highest mountain* does not have the corresponding reading for the same reason.

As summarized in Table 2, the combination of the three comparison set possibilities (i.e., the type of elided superlative clause under our hypothesis) and the three relative scope possibilities of Deg -*er* or -*est*, NP and V (the scope NP > V > Deg being independently excluded by the meaning of Deg) yields 9 logical possibilities, out of which 5 readings are available for superlatives in the absence of an overt domain of comparison:^22^ as we saw, 3 readings are excluded by the partitive condition on superlatives and one by the Sag–Williams scope generalization.

**Table 2:** Readings triggered by superlatives complementing intensional predicates (e.g., John wants to climb the highest mountain) under the superlative clause hypothesis

Interpretation of superlatives		Comparison set
absolute (upstairs) *de re* the *λ*y -*est* y d-high mountain John wants to climb t	DP > V	DP or reduced predicative clause (e.g., mountains)
absolute (downstairs) *de dicto* John wants to climb the *λ*y -*est* y d-high mountain	V > DP	
#absolute upstairs *de dicto.* #-*est* John wants to climb a d-high mountain	Deg > V > NP	
Cf. comparative: *John wants to climb a higher mountain* *than Mont Blanc (is).*		
(high) relative (upstairs) *de re* the d-high mountain -est [ ] John wants to climb a d-high mountain	DP > V	Clause containing high VP (e.g., aspirant climbers)
*(high) relative (downstairs) *de dicto* *John wants -est [ ] to climb a d-high mountain	V > DP	
(high relative) upstairs *de dicto* -est [ ] John wants to climb a d-high mountain	Deg > V > NP	
Cf. comparative: *John wants to climb a higher mountain* *than Mary wants to (climb).*		
#low relative upstairs *de re* #the d-high mountain -est [ ] John wants to climb a d-high mountain	DP > V	Clause containing low VP (e.g., climbers)
(low) relative (downstairs) *de dicto* John wants -est [ ] to climb a d-high mountain	V > DP	
#low (relative) upstairs *de dicto* #-est [ ] John wants to climb a d-high mountain	Deg > V > NP	
Cf. comparative: *John wants to climb a higher mountain* *than Mary climbs.*		

### Comparison of theories

As a variant of the movement theory, the superlative clause hypothesis inherits most virtues and problems of the movement theory as compared to the in situ theory. By incorporating the possibility of superlative clauses, it further presents some specific advantages. This section reviews the advantages of the superlative clause hypothesis in Sect. 3.2.1 and makes some suggestions about the open issues it shares with the other theories in Sect. 3.2.2.

#### Advantages of the superlative clause hypothesis

First, the superlative clause hypothesis inherits the advantages of the movement theory over the in situ theory. As mentioned at the beginning of Sect. 3, the derivation of upstairs *de dicto* reading is the clearest advantage of the movement theory over the in situ theory: this reading cannot be captured by the in situ theory without some stipulations that seem to mimic the movement theory.

Another important argument for the movement theory relies on island effects, as in (100).

(100) # John admires everyone who climbed the highest mountain. (Heim 1995/1999: 15)

(100) does not exhibit a relative reading under which John is compared to other admirers of climbers and identified as the most demanding one (i.e., the climbers admired by John) climbed a higher mountain than the climbers admired by the other (relevant) people); this fact follows from the movement theory (vs. the in situ theory) since this reading would require moving -*est* out of a complex DP island.

This type of explanation has already been proposed for comparatives, which exhibit the same kind of effects as illustrated in (101).

(101) John admires everyone who climbed a higher mountain than Mary {*does (admire)/did (climb)}.

The unacceptability of the reading under which Mary is compared to John as an admirer has been claimed to derive from island effects due to both the movement of -*er* and the movement of the degree operator in the comparative clause (Heim 1985, 2001; Kennedy 2002; cf. Charnavel 2015 about similar facts with *same*/*different*).

Under the superlative clause hypothesis, this explanation directly carries over to superlatives: island effects are correctly predicted due to both the movement of -*est* and the movement of the degree operator in the elided superlative clause. More generally, the superlative clause hypothesis correctly predicts that relative readings are subject to any constraint on movement.^23^

A further argument for the movement theory (against the in situ theory) noted by Heim (1995/1999) and inherited by the superlative clause hypothesis involves relative readings with arguments of transitive adjectives. In (102), the reading under which John is angrier at Mary than he is at anyone else can be captured by assuming -*est* movement as in (102)b using two-place -*est*.

(102)
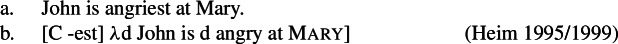

Finally, Bumford (2018) shows that sloppy readings of superlatives such as (103) provide a further argument for the movement theory. The problem for in situ analyses is that however the domain of comparison for the superlative is computed, the superlative has to quantify over the noun phrase ‘books with his name in the title’ where the pronoun is rigidly bound to John: only the strict reading is predicted to arise. Scope-taking theories of superlatives – including the superlative clause hypothesis – have no comparable difficulty because the superlative quantifies over a constituent large enough that the pronoun can be bound – strictly or sloppily – within it.

(103)

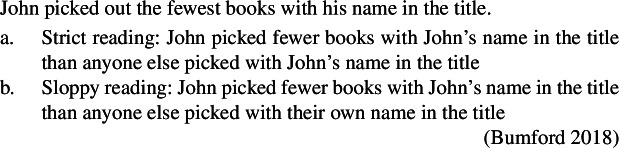

Second, the superlative clause hypothesis presents several conceptual advantages over the standard movement theory. The most important one, in my view, is that it analyses comparatives and superlatives in a parallel fashion (while integrating their intrinsic difference relative to partitivity). Beyond general parsimony, this provides a stronger motivation for -*est* movement: it is not only required for interpretive reasons, but also for reasons of ellipsis licensing, just like in the case of comparatives. Moreover, by equating the comparison domain with an (elided) clause, the superlative clause hypothesis eliminates some redundancy in the theory: first, the partitive condition is only expressed once in the definition of -*est*, while it is redundantly also expressed in the focus condition under Heim’s (1995/1999) approach; second, the superlative clause hypothesis suggests a way to treat absolute and relative readings (and NPI licensing under them) uniformly (by adjusting the elided clause, as shown in (99)), thus eliminating the duplication of lexical entries for -*est* (see fn. 14). Finally, the superlative clause hypothesis can analyze upstairs *de dicto* readings and sentences like (104) and (105) (cf. (89), (96)) uniformly.

(104) Mary_i_’s father tells her_i_ to work (the) hardest that anyone {does/tells her to}.

(105) John wants to climb the highest mountain that anyone {wants to climb/does}.

Under the superlative clause hypothesis, (104) and (105) involve partially elided clauses, while upstairs *de dicto* readings like (77) involve fully elided clauses. It can thereby directly account for the licensing of NPIs like *anyone* in the subordinate clause (104) and (105) following Howard’s (2014) account. By contrast, the standard movement theory does not have any explanation for the licensing of such NPIs in sentences like (104) and (105), as shown by Howard (2014) (see Sect. 2.2.2). In other words, if superlative clauses must be assumed to explain the licensing of NPIs in (104) and (105), why not assume the availability of superlative clauses across the board?

#### Open issues regarding definiteness

The superlative clause hypothesis can thus be seen as a more parsimonious version of the movement theory: not only does it inherit the virtues of the movement theory over the in situ theory, but it also presents some unique conceptual advantages. As acknowledged by Heim (1995/1999), the movement theory nevertheless faces some problems revolving around the definiteness issue, which motivate the in situ theory. As these problems are presumably inherited by the superlative clause hypothesis, the goal of this section is to examine them and suggest some solutions.

As noticed by Szabolcsi (1986), the movement theory requires interpreting the determiner as an indefinite determiner in LFs involving -*est* movement. This is the case for both semantic and syntactic reasons. Semantically, upstairs *de dicto* readings, for instance, require an indefinite interpretation of the gradable nominal: in the scenario discussed in (77), John wants to climb any 6000 m high mountain; his desire does not imply that there is only one such mountain (in his desire worlds or in the real world). Syntactically, moving -*est* out of a definite DP would violate island constraints (but see Davies and Dubinsky 2003 for a more nuanced view on extraction from DPs). This raises several questions that seem to undermine the movement theory: how can a definite article turn into an indefinite article after movement? How can it make the correct predictions in cases of ties or so-called sandwich scenarios (detailed below)?

First, it is important to note that as Szabolcsi (1986) observes, indefiniteness effects are independently supported: superlatives can appear in environments licensing only indefinites such as existential constructions in (106) under relative (vs. absolute) readings:

(106)
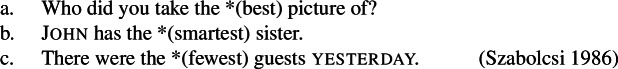

These effects are consistent with the movement theory and cannot be explained under the in situ theory. In fact, Sharvit and Stateva (2002: 486), which argue for an in situ theory, also assume that the definite determiner can be replaced by the indefinite determiner.

But it remains to explain why -*est* movement seems to turn the definite determiner into an indefinite instead of yielding ungrammaticality. To solve this problem, I hypothesize – inspired by Krasikova 2012 and Loccioni 2018, i.a. – that the overt definite article in superlatives need not mark definiteness of individuals (cf. adjectival *only*, see Sharvit 2015, i.a.). This is empirically supported by languages like French that can exhibit two definite articles (e.g., *la* *montagne* *la* *plus haute*, lit. ‘the mountain the highest’) and show mismatch in agreement between the definite article and the superlative adjective (e.g., *c’est parmi ses compagnes d’enfance qu’elle est* *le* *plus heureuse* ‘it is among her childhood friends that she is the_masc most happy_FEM’, Silberlight 1965). Conceptually, this further suggests a way of compositionally building the superlative on the comparative as hinted by morphology in languages after languages (see Bobaljik 2012). Recall that the only differences between two-place -*er* and two-place -*est* are the partitive condition in superlatives and the type of domain of comparison, which instead of containing just one element of comparison, contains all relevant elements of comparison. Following Krasikova (2012), I would like to suggest that the latter information is contributed by the (uniqueness presupposition of the) definite article (whose existential presupposition further indicates that the domain of comparison is not empty), which thus does not head a DP, but a DegP. Under this hypothesis, -*est* does not move out of a definite DP or violate islands to yield relative readings.

Although I will have to leave the details of this hypothesis for future research, we can more concretely assume that *the highest mountain* is ambiguous between [*the* [*the -est*] *d-high mountain*] and [*a* [*the -est*] *d-high mountain*] (which presumably does not give rise to redundancy due to *Maximize Presupposition!*, see fn. 25) and it is the complex quantifier *the -est* that can move to the propositional level under relative readings.

Furthermore, proponents of the in situ theory claim that indefiniteness makes incorrect predictions in some subtle scenarios involving ties. First, consider (107) in a scenario in which John and Bill climbed the same mountain, which is higher than mountains climbed by other people (see Heim 1995/1999: 13–14, Sharvit and Stateva 2002). This reading is captured by the in situ theory in (107)a, assuming that C is contextually restricted to mountains climbed by relevant climbers, or by the movement theory in (107)b based on two-place -*est* (cf. fn. 7).

(107)
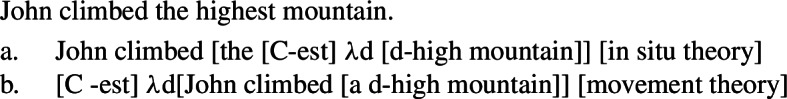

Under such a scenario, (107)a is predicted to be true, but (107)b is predicted to be false. Both Heim and Sharvit & Stateva agree that the judgments are not clear in such cases. That said, Sharvit & Stateva argue that (107) is not false, but at best misleading, which can only be explained under the in situ theory, if it is assumed that focus on *John* induces a (cancelable) implicature that the alternatives are false.

But conversely, the felicity of Heim’s (1995/1999) German example (108) remains unexplained under the in situ theory.

(108) Wenn niemand eindeutig den höchsten Berg besteigt, wird der Preis nicht vergeben. Lit. ‘If nobody unambiguously the highest mountain climbs is the prize not awarded.’ (Heim 1995/1999: 14)

This suggests that the relative reading can obtain under two construals of the domain of comparison (i.e., the relative construal *the highest mountain that anyone climbed* or the absolute construal *the highest mountain that there is* with contextual restriction to the mountains climbed^24^), which supports movement theories. To fully explain the variety in judgments, it remains to explain (perhaps based on a bias for truth) why for some speakers or/and in some examples, one LF is chosen over the other, which I have to leave for future research. But crucially, the movement theory, which overgenerates in some cases like (107), fares better than the in situ theory, which fatally undergenerates in cases like (108).

Another type of controversial scenario involves ties between mountains. For example, let’s consider a scenario where John climbed two 4000 m mountains, while the other climbers reached lower summits. In this scenario, (107)a is predicted to be neither true nor false (because there is no mountain that is highest), and (107)b is predicted to be true. According to Sharvit and Stateva (2002), many speakers hesitate when judging (107), thus corroborating the in-situ analysis; for speakers who judge (107) true, it can be assumed that one of John’s mountains can be ignored in the comparison set.

But assuming that the DP (vs. DegP) can in fact be definite or indefinite under the movement theory as I suggested above, we correctly predict truth (under the indefinite construal) or indeterminacy (under the definite construal), which may explain the variety in judgments. That said, it remains to explain why some speakers do consider the definite construal here but do not seem to do so in intensional contexts (where there is usually no single target element in the alternative worlds). As far as I can see, this question subsists in some form or other under all theories (for instance, Sharvit & Stateva have to assume that the definite can be replaced with the indefinite determiner in intensional contexts).

There is yet another case that is empirically problematic for the movement theory according to Sharvit and Stateva (2002), which they refer to as sandwich scenarios.^25^ It involves negative superlatives as in (109).

(109)
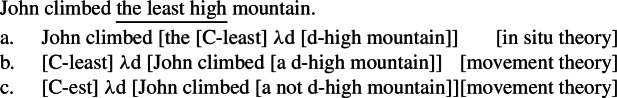

Consider a situation where John climbed a 3000 m high mountain, Bill climbed a 4000 m high mountain, and Mary climbed both a 2500 m high mountain and a 3500 m high mountain. Here, John’s mountain is ‘sandwiched’ between Mary’s mountains so that the person who climbed the lowest mountain (i.e., Mary) also climbed a higher mountain than another climber (i.e., John). According to Sharvit and Stateva (2002: 473), speakers judge (109) as false in this scenario (provided that the context makes clear that no mountain can be ignored). But one of the LFs predicted by the movement theory, namely (109)b, incorrectly predicts the sentence to be true: (109)b implies that there is a degree d such that everybody but John climbed a d-high mountain, which is the case of degrees between 3001 and 3500. The in situ theory, however, does not run into this overgeneration problem because it implies comparison between mountains.

Under the assumption that *least* is decomposable into the superlative morpheme -*est* and a negation (see Rullmann 1995; Stateva 2000; Heim 2006), the movement theory nevertheless correctly predicts the sentence to be false under the LF in (109)c as mentioned by Sharvit and Stateva (2002: 477): this LF implies that only John climbed a mountain that does not reach some degree of height; Mary’s lower mountain makes it impossible to satisfy. Furthermore, both types of LFs in (109)b and c are needed to explain the two types of upstairs *de dicto* readings with negative superlatives (Stateva 2000; Sharvit and Stateva 2002):

(110)
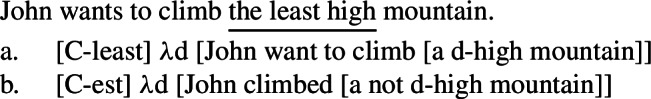

(110)a captures the ‘at least’ upstairs *de dicto* reading: (110) is true under this reading, e.g., in a scenario where to improve their ranking, John wants to climb a 3000 m high mountain (or higher), Mary wants to climb a 4000 m high mountain (or higher), and Bill wants to climb a 5000 m high mountain (or higher). (110)b captures the ‘at most’ upstairs *de dicto* reading: (110) is true under this reading, e.g., in a scenario where to remain safe, John wants to climb a mountain that is no higher than 3000 m, Mary wants to climb a mountain that is no higher than 4000 m, and Bill wants to climb a mountain that is no higher than 5000 m.

To derive all readings with negative superlatives under an in situ theory, Sharvit and Stateva (2002) must instead treat DPs in a nonstandard way in intensional environments (i.e., as properties), thus requiring ad hoc type shifters, and complex contextual restrictions of comparison sets (see details in Sharvit and Stateva 2002).

In addition, Büring (2007) observes that unlike the movement theory, the in situ theory undergenerates similar readings with comparatives. First, observe that in the same scenario as (109), (111) is judged false. This is correctly predicted by both the movement theory (under the LF with split scope of -*est* and negation) and the in situ theory (relying on comparison between mountains, not heights).^26^

(111) John climbed a less high mountain than Mary (did).

But Büring shows that the in situ theory makes wrong predictions when the *than*-clause contains an intensional predicate: for instance, it wrongly predicts the truth conditions of (112) to be as in (b).^27^

(112)
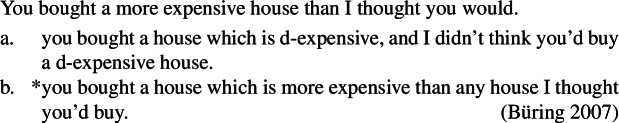

On the contrary, the movement theory makes the correct prediction as in (112)a and it further correctly predicts that (113) has two readings depending on the scope of the negation.

(113)

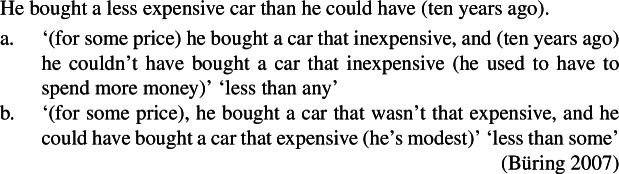

In sum, the in situ theory (based on individual comparison) can easily capture standard sandwich scenarios in both superlatives and comparatives, but crucially undergenerates some readings with intensional predicates (or requires extra and ad hoc machinery to generate them). Instead, the movement theory easily captures these readings, and only overgenerates in nonintensional sandwich scenarios. We thus reach the same outcome for all tricky cases involving definiteness: the movement theory definitely fares better than the in situ theory, which fatally undergenerates. Under the movement theory (including the superlative clause hypothesis), it only remains to understand why some predicted readings are unattested, which will probably require clarifying independent scope constraints with intensional predicates.^28^

## Conclusion

To conclude, both the case of intensional superlatives and that of upstairs *de dicto* readings provide support for the superlative clause analysis. The hypothesis that the domain of comparison can be syntactically represented by a degree clause derives the correct range of readings and the properties associated with them in both cases. In particular, it crucially entails the possibility of splitting the scope of the superlative morpheme and the measuring relation across intensional predicates, thus overcoming undergeneration problems of previous theories. At the same time, it implies specific constraints on the construal of the comparison domain due to degree quantification, which also avoids their overgeneration problems. Furthermore, the superlative clause hypothesis exclusively relies on independently motivated ingredients drawing from the theories of degree quantification, ellipsis, and scope.

One conspicuous argument has nevertheless recently been provided that could potentially challenge the empirical adequacy of the superlative clause hypothesis. Bumford and Sharvit (2022) observe that NPIs can be licensed outside superlative noun phrases, as illustrated in (114).

(114)
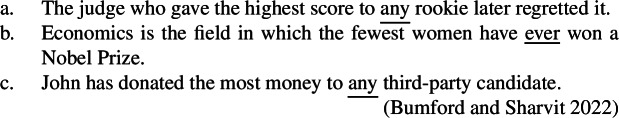

In these sentences, the NPIs are available only in the presence of a superlative and only under relative readings. Nevertheless, they do not occur in the restrictor of -*est*, even assuming ellipsis of the superlative clause.

(115)
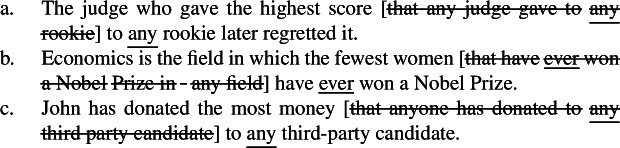

Contrary to what Bumford and Sharvit (2022: 270) claim, these NPIs are part of the comparison class: for example, the comparison in (115)a is not just between judges, but between pairs of judges and rookies; in fact, the NPIs do appear in the elided superlative clauses in (115). But their point remains that the overt NPIs occur outside it, which seems to challenge the predictions of our theory.^29^

But there is an alternative hypothesis consistent with the superlative hypothesis, which consists in assuming the presence of a higher implicit operator licensing NPIs in the presence of the superlatives. Several observations motivate this hypothesis. First, in all examples mentioned by Bumford and Sharvit (2022), the main variable element in the comparison class (e.g., the judge in (115)a) is expressed as the definite head of a relative clause (as in (115)a and c), as focused (as in (115)b) or as questioned (see Bumford and Sharvit 2022: 270); this is expected under the assumption that they are associated with a focus operator. Second, there is a rich literature independently demonstrating the need for covert focus operators to license NPIs (i.e., *only* and *even*; see Krifka 1995; Chierchia 2013, i.a.). Third, all examples require not only the superlative, but also a specific choice of predicates to license NPIs, as we will see. All this suggests that the NPIs in examples (115) are licensed by a covert *even* E (also associating with the main element of comparison) as illustrated in (116); the superlative does not directly license them, although it plays a crucial role.

(116) E [*John*_F_ has donated the *(most) money to *any*_F_ third-party candidate]

Specifically, the superlative changes the monotonicity of the environment hosting the NPI: while it is upward entailing in its absence ((117)b entails (117)a), it becomes nonmonotone in its presence, crucially only under a relative reading ((118)b does not entail (118)a, nor does (118)a entail (118)b).

(117)
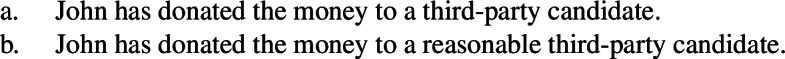

(118)
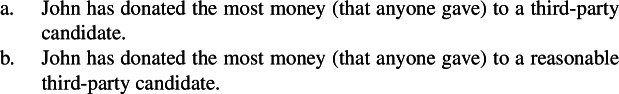

As has been shown by Crnič (2014), NPIs can be licensed in nonmonotone environments as long as the context of the sentence allows the presence of covert *even*, namely if alternatives are construed as more likely or expected, as required by *even*. The superlative also plays a crucial role in this respect by evoking the end of a scale, which interacts with the rest of the sentence to yield unexpectedness or scandalous effects (cf. Charnavel 2016). This point is easier to see in (115)b than (115)c, where John’s characteristics are unspecified in the lack of context. In (115)b (repeated below as (119)a), the unexpectedness arises from the combination of a low point induced by the superlative and the rest of the sentence creating an appropriate context (i.e., yielding the underlying message that women are wronged because it would be expected that they benefit from the same prestige as men – e.g., by winning a prestigious Nobel Prize); this is evidenced by the contrast with (119)b and (119)c, where either the superlative or the predicate have been changed, thus yielding infelicity.

(119)
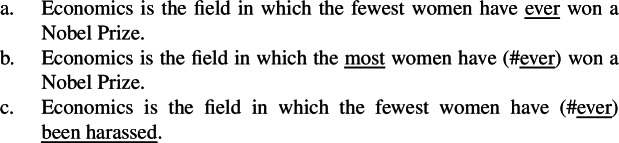

Further work is required to explain in detail all cases of NPIs appearing to arise outside the restriction of the superlative morpheme. But this preliminary investigation suggests that after all, such data do not necessarily challenge the empirical adequacy of the superlative clause hypothesis.^30^

Furthermore, it is not only in terms of empirical predictions, but also in terms of parsimony that the superlative clause hypothesis compares favorably to previous analyses by bringing closer the analyses of comparative and superlative constructions, which usually remain largely disconnected despite clear morphosyntactic similarities. Specifically, the hypothesis that comparative clauses have superlative counterparts opens the possibility of a unified theory of comparandum construal (cf. Gawron 1995) relying on degree clauses elidable to various extents. In this article, we have only argued that superlative clauses are attested at least in some cases. A stronger case for unification would require a systematic comparison of all possible types of comparandum in comparative and superlative constructions, which goes beyond the scope of this article (but see Appendix, Sect. A.2, for a discussion of two apparent discrepancies between comparative and superlative clauses). The few cases examined systematically in this article will hopefully motivate further research testing the underlying general hypothesis that comparative clauses have superlative counterparts, which promises to bring closer together the syntax/semantics of comparatives and superlatives.

## Appendix

### A.1 Refining the class of intensional superlatives (extension of Sect. 2.2.4)

As discussed in Sects. 2.1 and 2.2.4, the low reading of superlatives like *longest* does not have the same properties as the apparently low reading of other modifiers like *wonderful*, motivating the hypothesis that superlatives give rise to a specific LF (involving split scope of the superlative morpheme -*est* and the measuring relation) inducing this reading. But it has been observed that ordinals like *first*, adjectival *only*, and numeral-like modifiers like *few* also behave like intensional superlatives. Section 2.2.4 suggests that this observation can be explained by the hypothesis that all these elements can take a superlative clause (i.e., a clause denoting a degree-based comparison class). The goal of the present section is to provide more concrete suggestions for further research as to how the superlative clause hypothesis could extend to ordinals, *only* and numeral-like modifiers.

At first glance, ordinals seem most similar to superlatives since they involve ranking on a scale, although they do not lexically specify the type of ranking, which is usually some spatial or temporal ordering induced by pragmatically or syntactically determined context. As noticed by Bhatt (2006) and Bylinina et al. (2015), ordinals furthermore exhibit several properties characteristic of superlatives: they give rise to absolute and relative readings, their truth conditions can be influenced by focus, and they can take nonmodal subject infinitival clauses (as in (75)b and c). A reasonable analysis is thus to analyze the low reading of *first* just like *longest* in (32), assuming – based on etymology – decomposition of *first* into a superlative morpheme and *fore* (cf. Barbier 2007).^31^

Just as in (120)a, the clause in (120)b is construed as the domain of comparison complementing -*est*. Unlike in (120)a, however, something must be said about how exactly ordering of writing is here interpreted as the only relevant ordering. As noted by Heycock (2005), it seems that *fore* requires an argument specifying the type of degree property (fore with respect to what?). And as Sharvit (2010) shows, the ordering can in principle be contextually determined (by the way the books are stacked, for example). What will need to be spelled out for (120)b in further research is thus why and how the local verb *write* must play a crucial role in this specification.^32^ This point will also be crucially relevant to derive the high reading, where ordering seems to also be determined by material in the clause (the higher verb); in this respect, it will probably be important to recall that under our hypothesis, some of the so-called high readings are subclasses of the low reading (see Sect. 2.2.2). The other point to specify in future research will concern the extension of the analysis of *first* (or *last* similarly roughly decomposable as *late-est*) to nonextreme ordinals like *second* where an ordinal morpheme (*n-th*) must stand for -*est* in (120)b (see Alstott 2023 for a relevant discussion).

Numeral-like modifiers such as *many* or *few* and numerals like *two* have been the subject of many studies, which cannot be reviewed or evaluated here (see Rett 2018 for a review about the semantics of quantity words). But at least some parts of this literature reveal that they can also be conceived as similar to superlatives both in taking a domain argument and in being treatable in degree-based accounts. Specifically, the relevant quantity (its cardinality in the case of numerals like *two*, or its position with respect to some standard of quantity in the case of *many* or *few*) needs to be determined based on a set; in recent analyses, this set is a set of degrees denoted by the argument of quantity words (see references in Rett 2018). In our cases, we can thus assume that under the low reading, this set is explicitly expressed by the clause as roughly represented in (121).

In other words, the low reading of numerals can be assumed to derive from the construal of the clause as an amount relative. Amount relatives have also been the subject of numerous studies that go beyond the scope of this article (see Grosu and Landmann 2017 for a review), but many agree on treating them as degree clauses, which support the idea that our superlative clause hypothesis naturally extends to clauses modifying numerals.

Finally, what about *only*? *Only* is perhaps the topic of even more studies, which cannot be done justice here. But again, several properties liken *only* to superlatives.^33^ In fact, Heim’s (1995/1999) two-place lexical entry of -*est* in (33) is explicitly based on the semantics of *only* because both *only* and superlatives require a set of alternatives as argument, which can be determined by focus. Sharvit (2015) even proposes that adjectival *only* is the phonetic realization of -*est*. Furthermore, although this point remains debated, an important group of studies treats *only* as a scalar element, whose semantic contribution is not just to exclude some alternatives, but to exclude alternatives that are higher on a scale; recently, Greenberg (2022) treats *only* as the superlative antonym of *even*, in the sense that *only* presupposes that its prejacent is the weakest alternative in the relevant domain, while *even* presupposes that its prejacent is the strongest alternative. For our purposes, all this means that just as in the case of *longest*, the clause under the low reading can arguably be treated as explicitly denoting the set of alternatives C taken as argument by *only*. Under a scalar, superlative analysis of *only*, the role of *only* is to pick an endpoint of a scale in this set. Continuing the analogy, it is reasonable to further assume that the clause is also a degree clause, as the relevant scale in the case of the low reading is a quantitative one (i.e., John said that Tolstoy wrote one book, no more – vs. John said Tolstoy wrote a book of mediocre quality, not a better one):

Unlike Heycock’s account that only relies on the exclusive component of *only* (negating alternatives), this sketched proposal thus also builds on the scalar component of *only*.^34^ As in the case of ordinals and numerals, this suggestion would of course require much more investigation both for the cases at hand and for its consequences on the various existing debates about *only* in the literature.

### A.2 How to derive the lack of superlative counterparts to comparative subdeletion and comparative ellipsis (extension of conclusion)

The superlative clause hypothesis defended in this article relies on a parallel analysis of comparative and superlative constructions. To what extent can this hypothesis be generalized beyond the cases of intensional superlatives and upstairs *de dicto* readings detailed in the paper? As further support for the hypothesis, this section discusses two facts that could appear to challenge the hypothesized parallel between comparative and superlative clauses (i.e., the lack of superlative counterparts to comparative subdeletion and comparative ellipsis) and suggests that they can be derived by independent differences between comparatives and superlatives.

Comparative subdeletion and comparative ellipsis are illustrated in (123)a and (123)b.

In comparative subdeletion (vs. comparative deletion), only the degree variable is covert, the degree predicate (e.g., *large* in (123)a) remains overt (see Lechner and Corver 2017 for a review). In comparative ellipsis (cf. phrasal comparatives, see (2)), the comparative clause contains only a remnant (e.g., *Ben* in (123)b), which contrasts with a focused element in the correlate matrix clause (e.g., *Ann* in (123)b) (see Lechner 2020 for a review).

Strikingly, neither case seems to have a superlative counterpart, as shown in (124).

At first glance, this observation seems to challenge the superlative clause hypothesis under which we expect comparative and superlative clauses to be subject to the same types of ellipsis process. But I would like to suggest that on closer scrutiny, this discrepancy between comparatives and superlatives derives from their intrinsic difference, namely from the partitive condition on superlatives.

This is easiest to see in the case of subdeletion. Given the nonidentity between *long* and *large*, the definedness condition requiring that the correlate P belong to (and thus match) the set of comparison C is not satisfied. Subsuperlatives are thus unavailable because they violate the partitive presupposition.^35^

The argument is more indirect in the case of comparative/superlative ellipsis. In a nutshell, it has been shown that comparative ellipsis, just like comparative subdeletion, obligatorily exhibits coordinate-like properties, unlike other types of comparatives that only optionally do so (Corver 1993; Lechner 2001, i.a.). The exact reason why these structures require a coordinate parse remains debated, but it seems reasonable to tie this possibility to the relational nature of comparatives, i.e., comparatives relate two distinct elements. On the contrary, superlatives, which are partitive, cannot arguably have a coordinate parse, thus explaining the ungrammaticality of superlative ellipsis.

Specifically, Lechner (2001, 2004, 2020) provides several arguments showing that comparative ellipsis is not a specific ellipsis process, but results from the combination of comparative deletion (which he analyzes as head raising) and gapping, which crucially requires a coordinate parse. To provide a single example (see other arguments in Lechner 2001, 2004, 2020), comparative ellipsis structures, just like gapping structures, are constrained by isomorphism: the antecedent and the gap have to be embedded at the same depth inside their respective conjuncts, as illustrated in (125) for gapping and (126) for comparative ellipsis; (127) further shows that the same holds of partially reduced comparative clauses, thus corroborating that (126) is derived by gapping (vs. direct analysis mentioned in the introduction).

Strikingly, the same kinds of arguments have been provided by Corver (1993), among others, about subcomparatives. For instance, the contrast between (128)a and (128)b shows that subcomparative deletion requires a parallel, coordinate-like configuration, while that between (128)a and (128)c shows that comparative deletion does not.

Mismatch evidence has already shown that subsuperlatives violate the partitive condition on superlatives. I further suggest that the requirement of a coordinate parse provides the same argument, thus showing that both subsuperlatives and superlative ellipsis violate the partitivity condition.^36^

The incompatibility of a coordinate parse with superlatives may be due to the independent fact that entailment of a conjunct by the other is generally banned. Specifically, Hurford’s (1974) constraint has been shown to extend from disjunction to conjunctions (Katzir and Singh 2014, i.a.): for example, the conjunction in (129) is odd because the second conjunct entails the first one.

(129) #John lives in Paris and (he lives) in France.

Crucially, parsing a superlative as a conjunction would yield the same type of infelicity because of the partitive nature of superlatives as sketched in (130) (which purposely remains unspecified with respect to the contribution of -*est*).^37^

Due to their relational nature, comparatives, however, can perfectly be expressed as involving a conjunction as shown in (131).

This unavailability of a coordination parse in superlatives vs. comparatives can thus explain why unlike comparatives, ellipsis processes restricted to coordination structures cannot target superlatives. In fact, gapping and pseudogapping are also ruled out in superlatives unlike in comparatives:^38^

In sum, two facts that could appear to challenge the hypothesized parallel between comparative and superlative clauses in fact promise to be accounted for by independent differences between comparatives and superlatives. This supports our hypothesis that the comparandum can be expressed in the same way in comparative and superlative constructions modulo their intrinsic semantic differences.

## Footnotes

1 The same holds of comparatives involving measure phrases like (i), which will not be discussed in this article (see some discussion in Pancheva 2006, i.a.). Further note that interestingly for our general purposes here, Tomaszewicz-Özakin (2020) argues that the superlative counterpart of measure phrases does exist in some languages like Polish or Turkish as in (ii), and analyzes them as modifiers of the comparison set.

2 Unless a source is indicated, examples have been checked with a few native speakers.

3 Gawron’s (1992, 1995) goal is to treat uniformly some ambiguities found in both comparatives and superlatives. Specifically, he argues that both comparatives and superlatives have implicit degree clause restrictors partly determined by ellipsis (analyzed as higher order unification). In this respect, the present article clearly follows in its footsteps (even if it adopts a different view on ellipsis). However, Gawron’s (1992, 1995) main empirical basis differs from that of the present paper, which concentrates on various kinds of interaction between superlatives and intensional predicates (but see Sect. 3.1.2 for a discussion of one directly relevant example by Gawron in (92)).

4 The term ‘correlate’ is usually used to refer to the individual in the matrix clause (e.g., *Ann* in (1)) that contrasts with the individual in the standard of comparison (e.g., *Ben* in (1)). I here use the term a bit differently by referring to the whole clause in which the degree head appears (e.g., *Ann is tall* in (1)).

5 The mechanism for recovering the adjective (*long*) and the noun (*book*) in the comparative clause is debated. In predicative comparative constructions, which have been more extensively discussed, the adjective is often assumed to be recovered by ellipsis (comparative deletion, as in Kennedy 2002, i.a.) or sometimes by movement (head raising, as in Lechner 2004, i.a.). The few analyses of attributive comparative constructions (Kennedy and Merchant 2000; Lechner 2004, i.a.) also assume movement or/and ellipsis of the adjective and the noun under some additional assumptions about the structure that cannot be detailed here. Note that another important topic for debate in the comparative literature, which will not be addressed here either, is the way the adjective, the noun, and the comparative clause combine (see review in Lechner and Corver 2017; Lechner 2020). Heimian analyses, which underlie most hypotheses mentioned in the main text, rely on the hypothesis that the comparative morpheme forms a constituent with the comparative clause.

6 Following Heim (1995/1999), Howard (2014) does not treat C as a set of degree sets (like Romero 2013), but as a set of intensions thereof (i.e., a set of degree properties) because of cases involving ties. For instance, Howard (2014: 24) claims that *Mary sung the loudest* is not judged true if Mary and Lee sang at exactly the same intensity; this is only captured under the degree properties hypothesis: under the degree sets hypothesis, the set of degrees such that Mary sung to those degrees is indistinguishable from the set of degrees such that Lee sung to those degrees. Conversely, Romero (2013) adopts degree sets because modal superlatives allow, in fact require, the possibility of ties between possible worlds: *Mary is the prettiest possible* does not preclude Mary to be as pretty in some possible worlds as she is in the actual world (see discussion in Loccioni 2018: 86–87; see also Sect. 3.2.2 for some discussion about scenarios with ties).

7 For independent reasons related to indexical binding, Bassi (2021: 136) similarly proposes that subordinate clauses combining with *only* can be duplicated, with one being elided under identity (e.g. *I am the only one*  *who submitted x’s paper*).

8 I here gloss over various possible derivations depending on where the head *book* is interpreted (cf. Hulsey and Sauerland 2006): *book* can be interpreted both within and outside the clause, in which case the speaker and John agree that it is a book (as entailed by my paraphrase in the main text), or only within the clause (as in (38)b). See Sportiche (2016), i.a., for reconstruction effects and their derivation in relative clauses. What matters here is that *book* is interpreted at least low, -*est* is interpreted only high, and *d-long* only low. Further note that I do not here detail the syntactic structure, esp. the highly debated structure of the degree head projection (see fn. 6).

9 As mentioned by an anonymous reviewer, the elided clause cannot, however, be overtly expressed in such constructions in English: for example, *the longest book that Tolstoy wrote that Tolstoy wrote* is interpreted as a redundant stacked relative clause. Although the exact reason for this remains unclear, note that this is reminiscent of obligatory deletion in comparative clauses under maximal identity (e.g., **Pico’s novel is more interesting than Brio’s novel is interesting* in Kennedy and Merchant 2000: 96). Further note that just as in the case of comparative clauses (see Kennedy 2002, i.a.), the elided clause cannot be identical to a previous clause (e.g., *Tolstoy wrote a 1000-page long book. John read a longer book than Peter* (**wrote*)); in the case of superlatives, this would anyway violate the partitive condition.

10 Sharvit (2007) is the published version of Sharvit’s (2003) handout, where she presents a different final analysis: while Sharvit (2003) argues for split scope, Sharvit (2007) argues for reconstruction of -*est* (like Hulsey and Sauerland 2006: (53)e) mainly due to the problem discussed here. As such, she nevertheless inherits most of the problems of reconstruction accounts, and furthermore relies on Hulsey & Sauerland’s assumptions that the adjective *long* can be interpreted in an intermediate position on the edge of the finite CP, and that -*est* can be interpreted between the *λ*-operator and this intermediate trace.

11 A precise examination of the semantics of *say* would lead me too far afield, but note that promisingly, the low reading seems to require a scare quote intonation when an inanimate subject forces a nondoxastic interpretation and only a comparative (vs. quantitative) judgment is expressed as in (i). Recall from Sect. 2.1.1 that this intonation signals that it is not an actual low reading involving a superlative clause.

12 In an unpublished version of Sharvit (2007) closer to Sharvit (2003), Sharvit makes a concrete proposal for intensionalized 3-place -*est*. Note that following Howard (2014), we have similarly intensionalized two-place -*est*, for independent reasons, namely partitivity and cases of ties (see fn. 7).

13 The problem of NPI licensing with relative readings identified by Howard may thus be solved without having to postulate homonymy between -*est* for relative readings (Heim’s lexical entry in (i)) and -*est* for absolute readings (von Fintel’s lexical entry in (ii)) as in Howard (2014) − and without failing to derive the upstairs *de dicto* reading as in Bumford and Sharvit 2022 that propose a dynamic take on superlative semantics to solve the NPI licensing problem with relative superlatives.

All cases can arguably be captured using (i), where the environment licensing NPIs is the domain argument, i.e., the superlative clause C (cf. the NP Q in (ii)) because it is Strawson downward entailing.

14 The superlative clause hypothesis also straightforwardly derives the readings observed with other NPIs: for example, the comparison set in (i) involving *anyone* is created through quantification over individuals, as shown in (ii).

Multiple NPIs are also correctly predicted to create sets through multiple quantification (e.g., over times and individuals in the presence of both *ever* and *anyone*; see Howard 2014).

15 Heycock (2019) also shows that the contrast reported by Hulsey and Sauerland (2006: 116) in (i) regarding extraposition cannot be used as reliable evidence. According to Hulsey & Sauerland, extraposition, which excludes a raising analysis of relative clauses, is incompatible with low *ever* as predicted by Bhatt. But the small-scale questionnaire presented by Heycock (2019: 103–106) casts doubt on this contrast as it does not reveal the contrasts predicted by Hulsey and Sauerland (2006) regarding extraposition.

16 The other (less accessible) reading is a reading under which the clause denotes the domain of comparison (and thus licenses *ever*) and is extraposed, while *in the list* modifies book. See fn. 16 on extraposition.

17 This may provide an argument for the hypothesis that superlatives only have degree-based domains of comparison (vs. individual-based domains of comparison, cf. debate on phrasal comparatives; see Tomaszewicz-Özakin 2020, i.a.) as already suggested in fn. 14. Further arguments should bear on whether *of*-phrases (as in e.g., *the highest of any island*) can be interpreted as sets of degree (i.e., roughly as *the highest mountain that* *λd any island has*  ). See some relevant discussion at the end of Sect. 3.1.2.

18 Charnavel (2015: 133–137) argues that the hypothesis of such underspecified elements is independently supported by the behavior of relational nouns like *enemy* of *neighbor*, or the French clitic *se*.

19 I have so far adopted the standard lexical entry of two-place -*est* according to which P denotes a set of degree properties, while C denotes a set thereof (see (16) or (33)). But note that we could alternatively assume that C also denotes a set of degree properties as long as the standard of comparison includes an existential quantifier (inducing universal quantification under the negation contributed by -*est* – that would incidentally suggest an explanation as to why it can take the overt form of a negative polarity item). This further likens the lexical entry of two-place -*est* and that of two-place -*er*:

To avoid contradiction in the assertion, this would require restricting the domain of quantification of the existential quantifier so as to exclude John, which can be ensured by contrastively focusing *John*. Note that the same happens in comparatives such as *John did better than* *anyone* (vs. *John did better than* *anyone else*) or *John did better than* *ever*.

20 The *de re* vs. *de dicto* distinction does not usually qualify the same elements in the literature on comparatives and superlatives: *de dicto* (vs. *de re*) is usually intended to characterize the DP (e.g., *the*/*a* *d-high mountain*) in the superlative literature, but the *than*-clause in the comparative literature. Furthermore, the scope of the DegP with respect to the intensional predicate is indicated using a specific term *upstairs* (vs. *downstairs*) in the superlative (vs. comparative) literature. I will henceforth stick to the superlative terminology, complementing it with the *low* vs. *high* contrast to capture differences in ellipsis size, and the degree *de re* vs. degree *de dicto* contrast to indicate DegP scope.

21 Charnavel (2024) however, claims that this reading exists, and among the speakers I consulted, a few speakers can marginally get this reading. This may be due to the fact that under some conditions, properties may be read *de re* (cf. *de qualitate* readings in Schwager 2009, see also Teodorescu 2009) as illustrated in (i) from Teodorescu (2009: 73).

This sentence can be true (assuming that red tomatoes and juicy tomatoes are coextensive) under a scenario where Fred is blind and all he wants to eat is a juicy tomato (but crucially, no specific one): *red* can here seemingly be read *de re* (see Schwager 2009 for a more precise discussion). It is conceivable that the superlative morpheme (e.g., *the most* in (92)) can marginally behave like *red* here (under some specific intonation). Further note that this could also explain why *John wants to climb the highest mountain* can marginally have an absolute upstairs *de dicto* reading where all John wants to climb is any mountain that is at least 8700 m high, and he does not know that only Mount Everest qualifies (see discussion later in the main text). Thanks to an anonymous reviewer for bringing up *de qualitate* readings to my attention.

22 There are even more readings if we take into account the *de re* vs. *de dicto* distinction in the comparison set (comparative/superlative clauses can be *de re* or *de dicto* under low scope of DegP), *de qualitate* readings (see fn. 22) and the distinction between *de dicto* and *de credito* readings (see Yanovitch 2011, i.a.).

23 For instance, example (i-a) (suggested by an anonymous reviewer) is correctly predicted to be unacceptable due to *for*-trace effects, just like (i-b) and (i-c).

Similarly, Heim (1985) is not definitive about the exact type of constraints -*er* and -*est* are subject to, because the generalization proposed by Szabolcsi (1986) implies tighter restrictions on relative readings than island restrictions. For example, Szabolcsi (1986) claims that (ii) does not exhibit a relative reading where the comparison is made with respect to the subject of *say* (i.e., ‘who claimed for a smaller n than anyone else did that you got n letters?’, Heim 1995/1999: fn. 26).

The judgments about this type of sentence seem to me to be less clear than about sentences like (100) involving islands. This is consistent with the hypothesis that movement of -*er* and -*est* is similar to Quantifier Raising (Heim 2001) where clausemateness restrictions are debated (see Farkas 1981 vs. Fox 2000, i.a.).

24 Under the analysis of the absolute reading that we suggest (with two-place -*est* scoping within the NP as in (99)), both the definite and the indefinite construal yield the same truth and definiteness conditions. By *Maximize Presupposition!*, the definite construal is thus chosen. But the availability of the indefinite construal is necessary under syntactic conditions that do not license a definite article. For example, Sharvit & Stateva observe that *Who did you sell the most expensive picture of?* does have an absolute reading, thus arguing against the strict correlation between indefiniteness and comparative readings proposed by Szabolcsi (1986). This is correctly predicted by our hypothesis.

25 Sharvit and Stateva (2002) (cf. Farkas and Kiss 2000) consider (i) as a further argument against the movement analysis. According to them, (i) does not yield a relative reading; this is expected under the in situ theory because the PP directly constrains the choice of the comparison set; but this does not (straightforwardly at least) follow from the movement theory. I agree that the PP is indeed preferably construed as the comparison set, thus yielding an absolute reading; but a relative reading is in fact available (cf. Coppock and Beaver 2014: fn. 14) if the context favors a construal where *in Europe* is not interpreted as the comparison set, e.g., because the common ground entails that only European cities are under discussion, or because they are contrasted with other cities; such interpretations can be favored by continuations such as “his friends only visited small cities in Europe”, or “Mary visited the largest city in Asia”.

26 Sharvit and Stateva (2002: 501) claim that the judgments for comparatives in sandwich scenarios are unclear based on the following sentence (in a scenario where Mary’s mountain is sandwiched between John’s mountains in terms of slipperiness):

Büring (2007) attributes the unclarity of judgments to the pragmatic oddness of their example, and instead uses the following example (in a scenario where Bob’s method is sandwiched between Mary’s methods in terms of expensiveness):

This suggests that the pragmatic orientation of the scale may matter (as in stereotypical context, it’s better to know the least expensive method, but to climb the most slippery mountain).

27 Partly due to the uncertainty of their judgments in sandwich scenarios (see fn. 27), Sharvit and Stateva (2002) do not extend their semantics of superlatives to comparatives: in particular, they treat the *than*-clause as a set of degrees, while they treat the domain of comparison for superlatives as a set of individuals. As mentioned by Büring (2007: fn. 3), it remains to be seen if Sharvit & Stateva’s solution for upstairs *de dicto* readings could extend to cases like (112) (which they do not explore).

28 For example, Sharvit and Stateva (2002) claim that the scope option where both negation and -*est* move, which is required to generate the ‘at least’ reading with intensional predicates (see (110)a), overgenerates a reading in sandwich scenarios. Another way to state the problem is to ask why the negation can move above the intensional predicate in (110), which may relate to neg-raising issues.

29 (115)a and c lend themselves to an analysis involving right node raising, just as can be the case in comparative clauses. Under Lechner’s (2004: 143) analysis combining right node raising with gapping, the overt NPI would be in the comparative clause, thus in the restrictor of the comparative morpheme as shown in (i).

But this implies the availability of gapping with superlative clauses, which is argued against later on in the Appendix (Sect. A.2). Furthermore, this analysis is unapplicable to example (115)b.

30 Further note that the alternative theory advocated by Bumford and Sharvit (2022) to derive these facts cannot derive upstairs *de dicto* readings (as they acknowledge, and as mentioned in fn. 14).
